# Highly variable chloroplast genome from two endangered Papaveraceae lithophytes *Corydalis tomentella* and *Corydalis saxicola*

**DOI:** 10.1002/ece3.7312

**Published:** 2021-03-19

**Authors:** Fengming Ren, Liqiang Wang, Ying Li, Wei Zhuo, Zhichao Xu, Haojie Guo, Yan Liu, Ranran Gao, Jingyuan Song

**Affiliations:** ^1^ Institute of Medicinal Plant Development Chinese Academy of Medical Sciences & Peking Union Medical College Key Lab of Chinese Medicine Resources Conservation State Administration of Traditional Chinese Medicine of the People's Republic of China Beijing China; ^2^ Medicinal Biological Technology Research Center Chongqing Institute of Medicinal Plant Cultivation Bio‐Resource Research and Utilization Joint Key Laboratory Sichuan and Chongqing Chongqing China; ^3^ College of Pharmacy Heze University Heze China; ^4^ Engineering Research Center of Chinese Medicine Resource Ministry of Education Beijing China; ^5^ Wuhu Institute of Technology Wuhu China

**Keywords:** chloroplast genome, *Corydalis saxicola*, *Corydalis tomentella*, Papaveraceae, taxonomic study

## Abstract

The increasingly wide application of chloroplast (cp) genome super‐barcode in taxonomy and the recent breakthrough in cp genetic engineering make the development of new cp gene resources urgent and significant. *Corydalis* is recognized as the most genotypes complicated and taxonomically challenging plant taxa in Papaveraceae. However, there currently are few reports about cp genomes of the genus *Corydalis*. In this study, we sequenced four complete cp genomes of two endangered lithophytes *Corydalis saxicola* and *Corydalis* *tomentella* in *Corydalis*, conducted a comparison of these cp genomes among each other as well as with others of Papaveraceae. The cp genomes have a large genome size of 189,029–190,247 bp, possessing a quadripartite structure and with two highly expanded inverted repeat (IR) regions (length: 41,955–42,350 bp). Comparison between the cp genomes of *C. tomentella*, *C. saxicola*, and Papaveraceae species, five NADH dehydrogenase‐like genes (*ndh*F, *ndh*D, *ndh*L, *ndh*G, and *ndh*E) with *psa*C, *rpl*32, *ccs*A, and *trn*L‐UAG normally located in the SSC region have migrated to IRs, resulting in IR expansion and gene duplication. An up to 9 kb inversion involving five genes (*rpl*23, *ycf*2, *ycf*15, *trn*I‐CAU, and *trn*L‐CAA) was found within IR regions. The *acc*D gene was found to be absent and the *ycf1* gene has shifted from the IR/SSC border to the SSC region as a single copy. Phylogenetic analysis based on the sequences of common CDS showed that the genus *Corydalis* is quite distantly related to the other genera of Papaveraceae, it provided a new clue for recent advocacy to establish a separate Fumariaceae family. Our results revealed one special cp genome structure in Papaveraceae, provided a useful resources for classification of the genus *Corydalis*, and will be valuable for understanding Papaveraceae evolutionary relationships.

## Graphical abstract

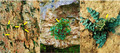

## 1 INTRODUCTION

Chloroplasts (cp), generally considered to have originated from ancient cyanobacteria, are the main site of photosynthesis and energy conversion in plant cells, containing the major enzyme systems for photosynthesis and a highly conserved genome (Ahlert et al., 2003; Moore et al., 2010). With the development of high‐throughput sequencing technology, cp genomics has made rapid progress (Li et al., 2015). The National Center for Biotechnology Information (NCBI) database included 377 complete cp genome sequences in 2010 and had more than 10,381 sequences in 2020 (https://www.ncbi.nlm.nih.gov/genome/browse/), a nearly 30‐fold increase over 10 years. Currently, cp genomics research is an intense area of botanical and genomic study.

Correct understanding of the relationship between different biological groups is the main focus of phylogenetic biology, the basis of taxonomy and naming, and a foundation for research in other branches of biology (Chen et al., 2016). Compared with traditional molecular markers, the cp genomes provide specific advantages for establishing plant phylogenetic relationships and taxonomic research (Guo et al., 2017). The length of cp genomes is usually 115–165 kb, a modest size that is easily sequenced. The longer sequence provided more sufficient information for phylogenetic analysis. Relatively conserved gene sequences allow produce co‐linearity among plant groups, and the evolution rates of coding regions and noncoding regions are significantly different to be suited for phylogenetic analysis of different ranks (Clegg et al., 1994). Taxonomists have used cp genomes to study plant phylogenetics and advocated for use of cp genomes as a super DNA barcode for species identification (Guo et al., 2017).

In recent years, a large number of cp genome have been sequenced, providing abundant data that can be used for plant phylogeny research to more accurately reveal the true evolutionary relationships between species and effectively solve difficult phylogenetic relationship problems in the study of complex plant taxa (Guo et al., 2019; Jansen et al., 2006; Zhang et al., 2017). Cp genomes have been successfully used as a “super barcode” to identify many taxonomically difficult species (Cui et al., 2019; Ying et al., 2019). With the reduced cost of sequencing and the development of bioinformatics technology, cp genome will be extensively used in future studies of plant taxonomy.

*Corydalis* DC., the largest genus of Papaveraceae, is recognized as one of the most taxonomically challenging plant taxa (Magnus et al., 1996). It has extremely complex morphological variation because of typical reticulate evolution and intense differentiation during evolution (Wu et al., 1996). Taxonomic study of the genus on the basis of morphological characteristics has been very difficult (Lu et al., 2018). Cp genomes have been proven effective for phylogenetic research of many taxonomically complex taxa. However, there currently are few reports about cp genomes of the genus *Corydalis*, but see two plants, *Corydalis trisecta* and *Corydalis conspersa* (Kanwal et al., 2019; Wu et al., 2020). Therefore, it is necessary to sequence the cp genomes of *Corydalis* plants in order to provide more accurate basis for the classification and identification of this genus.

**FIGURE 1:**
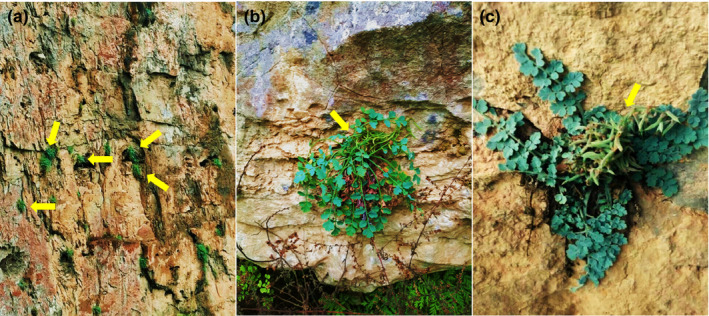
The habitat of *C. saxicola* and *C. tomentella*. (a) The distant view of steep cliff growing *C. saxicola*; (b) the close shot of *C. saxicola*; (c) the close shot of *C. tomentella*. The yellow arrows indicated the *Corydalis* plants

In this study, high‐throughput sequencing and comparative genomics were used to study the cp genomes of two *Corydalis* plants: *Corydalis saxicola* and *Corydalis tomentella*. They belong to Sect. *Thalictrifoliae* Fedde of the genus *Corydalis*, which grows in dry cracks of limestone (Figure 1) and is known as lithophytes. There are little available soil and water on the limestone, so they have been subjected to extreme environmental conditions, such as high temperature, drought, and high PH (Ren et al., 2019). Then, we asked whether the cp genome structures of these two lithophytes had special variation under the extremely harsh lithophytic environment, and whether these variation would affect their classification and identification. We sequenced four complete cp genome sequences from these two plants, described their genomic characteristics, conducted comparisons between these genomes and other Papaveraceae cp genomes, and analyzed the phylogenetic relationships on the basis of common protein CDS. Our study aim was to assess structural variation, and provide valuable resources for classification of the genus *Corydalis*.

## 2 MATERIALS AND METHODS

### 2.1 Materials, DNA extraction and sequencing

Plant materials were provided by the Chongqing Institute of Medicinal Plant Cultivation (CQIMPC) and identified by researcher Zhengyu Liu as *C. tomentella* Franch. and *C. saxicola* Bunting. The voucher specimens of the two species were deposited in CQIMPC, and the specimen accession numbers were *NC‐CQIMPC201651*, *NC‐CQIMPC201652 NC‐CQIMPC201661*, and *NC‐CQIMPC201662*, respectively. We collected young leaves from selected plants that were vigorous, healthy, and disease‐free. These leaves were wiped with 70% alcohol and repeatedly washed with sterile water before genomic DNA extraction. Total DNA was extracted using a Tiangen plant genomic DNA extraction kit (Tiangen Biotech Co.), and the DNA quality and concentration were detected using 1% agarose electrophoresis and a Nanodrop 2000. The DNA was sheared to yield approximately 500 bp long fragments for paired‐end library construction. The library was sequenced on Illumina HiSeq 4000 Platform (Illumina) according to the standard protocol of manufacturer's manual. Approximately 3–5 Gb raw paired‐end reads (2 × 150 bp) were obtained for each specimen.

### 2.2 Genome assembly and annotation

The cp genome were assembled on a Linux system. First, raw sequencing data were filtered using Trimmomatic (Version 0.36) to get the high‐quality clean data (Bolger et al., 2014). In the second step, we used the thirteen chloroplast genome sequences of Papaveraceae species which were downloaded from GenBank to establish a Basic Local Alignment Search Tool (BLASTn) database. Then the clean data were mapped to the BLAST database, and the mapped reads which were considered as reads from chloroplast genome were extracted. Next step, the extracted reads were assembled to contigs using SOAPdenovo2 (Luo et al., 2012). At last, SSPACE was used to construct the scaffold of the chloroplast genome (Boetzer et al., 2011), and GapCloser was used to fill gaps (Luo et al., 2012). The completed genomes were annotated using CPGAVAS2 (Shi et al., 2019), and the results were modified for starter and terminator revisions by Apollo software (Lee et al., 2009). CPGAVAS2 software was used to convert revised GFF3 format annotation results into a sqn format for NCBI submission. Sequin software was used to check and correct unsatisfactory comments in the sqn file, and the corrected results were submitted to the NCBI database. Physical maps of the cp genomes were drawn by GenomeDRAW (Marc et al., 2013) using a GB format file exported from the sqn file by sequin software.

### 2.3 Genome structure analyses and genome comparison

GC content was analyzed using MEGA6.06 software (Tamura et al., 2013). The distribution of codon usage was investigated using CodonW software with the RSCU ratio (Sharp & Li, 1987; Zhou et al., 2017). MISA software (http://pgrc.ipk‐gatersleben.de/misa/) was used to detect simple sequence repeats (SSRs) (MISA‐Microsatellite Identification Tool, 2017). Parameters were set as follows: no less than 8 single‐base repeat units; no less than 4 units with 2, 3 bases in one unit; and no less than 3 units with 4, 5, 6 bases in one unit (Huang et al., 2020). Tandem Repeats Finder v4.0.4 software (Benson, 1999) was used to detect tandem repeat sequences, and the default parameter was set to 2‐7‐7 ‐80‐10‐50‐500‐f‐d‐m (Li et al., 2014). REPuter software (http://bibiserv.techfak.uni‐bielefeld.de/reputer) was used to detect scattered repeating sequences (>30 bp) using the parameter: hamming distance = 3 (Stefan et al., 2001). VISTA software was used to compare multiple cp genomes (Frazer et al., 2004).

### 2.4 Phylogenetic analysis

A total of 13 cp whole genome sequences were used in cluster analysis. Eleven genomes were from Papaveraceae (*C. tomentella* MT093187 MT077878, *C*. *saxicola* MT077878 MT077879, *Papaver somniferum* NC029434, *Papaver orientale* NC037832, *Papaver rhoeas* MF943221, *Meconopsis racemosa* MH394401 NC039625, *Macleaya microcarpa* NC039623, and *Coreanomecon hymenoides* NC031446), and *Coptis chinensis* (NC001879) and *Nicotiana tabacum* (NC036485) genomes were included as outgroups. Of the Papaveraceae genomes, four genomes were newly sequenced in this study, and nine genomes were downloaded from the NCBI database. Common protein coding sequences were extracted from the cp genome sequences (Li et al., 2014), and multiple global alignments of the protein coding sequences was performed using the Clustalw module in MEGA6.06 software. Maximum‐Likelihood (ML) phylogenetic tree was constructed by MEGA6.06 software (Tamura et al., 2013). The program operating parameters were set as follows: a Tamura–Nei nucleotide substitution model with 1,000 bootstrap repetitions, accompanied by Gamma distributed with invariant site (G + I) rates, and partial deletion of gaps/missing data. The model with the highest bootstrap values at each node was determined to be the most appropriate model.

## 3 RESULTS

### 3.1 Chloroplast genomes features

**FIGURE 2:**
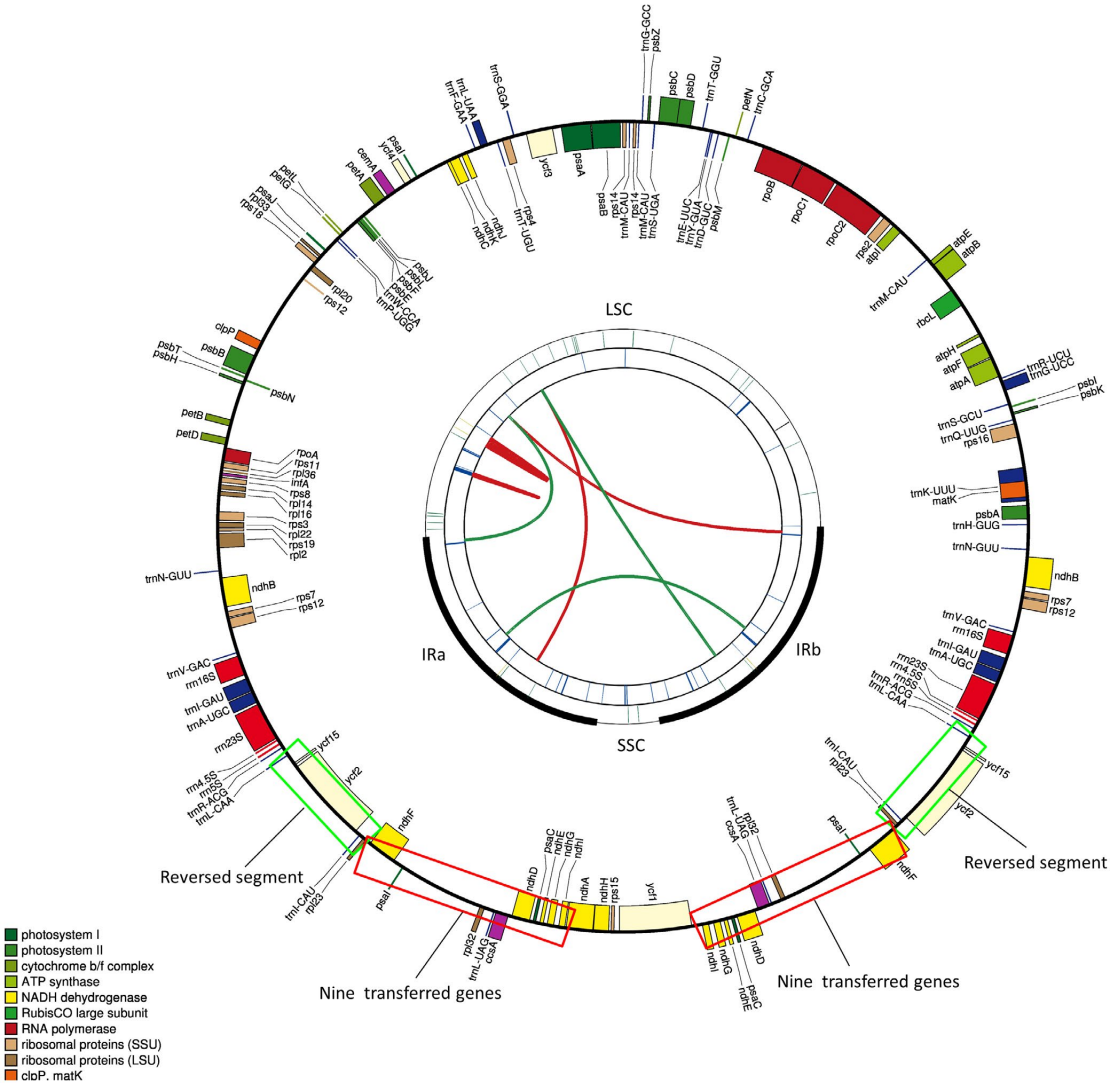
Schematic representation of the chloroplast genomes of *C. tomentella*. The map contains four rings. From the center going outward, the first circle shows forward and reverse repeats connected with red and green arcs, respectively. The next circle shows tandem repeats marked with short bars. The third circle shows microsatellite sequences identified by MISA. The fourth circle is drawn using drawgenemap and shows the gene structure of the plastome. The genes are colored on the basis of their functional categories. Genes inside and outside of the circle are transcribed in clockwise and counterclockwise directions, respectively. IR, inverted repeat; LSC, large single copy; SSC, small single copy. The red rectangles indicated the nine gens (*ndh*F, *ndh*D, *ndh*L, *ndh*G, *ndh*E, *psa*C, *ccs*A, *rpl32*, and *trn*L‐UAG) normally located in the SSC region have migrated to IRs; the green rectangles indicated the reversed segment involving five genes (*rpl*23, *ycf*2, *ycf*15, *trn*l‐CAU, and *trn*L‐CAA)

Approximately, 5.12, 5.23, 2.68, and 2.77 Gb raw paired‐end reads (2 × 150 bp) were obtained from the Illumina HiSeq 4000 Platform for MHJ‐1, MHJ‐2, YHL‐1, and YHL‐2, respectively. The raw sequencing data were filtered using Trimmomatic, 4.54, 4.61, 2.25, and 2.30 Gb of clean data were used to assemble the complete chloroplast genome. The complete *C. tomentella* genomes were 190,198–190,247 bp long and exhibited a typical angiosperm circular cp structure, containing four regions: large single‐copy region (LSC: 96,530–96,701 bp), small single‐copy region (SSC: 9,636–9,664 bp), and a pair of inverted repeats (IR: 41,955–42,002 bp) (Figure 2). The GC content of the genome and each genomic region was also typical of angiosperm cp structure. Specific lengths and contents are shown in Figure 2 and Table 1. The lengths of the two complete *C. saxicola* genomes were 189,029 and 189,155 bp, which were slightly smaller than those of *C. tomentella*. The cp genome structure, size of each region, and GC content were similar between the two species (Table 1).

**TABLE 1:** Summary of chloroplast genome features of *C. tomentella* and *C. saxicola*

Species	Voucher No.	Genbank No.	Total	Length (bp)			GC content (%)			
				IR	LSC	SSC	Total	IR	LSC	SSC
*Corydalis tomentella*	MHJ1	MT093187	190,247	41,955	96,701	9,636	40.3	42.2	39.2	35.4
	MHJ2	MT077878	190,198	42,002	96,530	9,664	40.2	42.2	39.0	35.4
*Corydalis saxicola*	YHL1	MT077877	189,155	42,350	94,744	9,711	40.2	42.2	39.1	35.1
	YHL2	MT077879	189,029	42,164	94,993	9,708	40.3	42.2	39.1	35.1

CPGAVAS2 was used to annotate the cp genomes of *C. tomentella* and *C*. *saxicola*. Removing duplicate genes, a total of 119 annotated genes (Figure 2, Table 2 and Table S1), including 78 protein‐coding genes, 37 tRNA genes, and four rRNA genes, were identified from the *C. tomentella*. There were 28 genes in the IR region, of which 15 were involved in gene expression. Introns greatly affect regulated selective splicing in the genome. There were 19 genes that contain introns in the *C. tomentella* cp genome. Most intron genes contained only one intron, while the *ycf*3 gene contained two introns. There were 12 introns with a length of more than 700 bp, and the longest gene was *trn*K‐UUU with a length of 2,478 bp. The gene features of *C. saxicola* cp genome were similar to those of *C. tomentella*. The *C. saxicola* cp genome contained 120 genes, including 78 protein‐coding genes, 38 tRNA genes, and four rRNA genes. Nineteen genes contained introns. The longest intron gene in the *C. saxicola* cp genome was *trn*K‐UUU, and its length was also 2,478 bp (Figure 2, Table 2 and Table S1).

**TABLE 2:** List of genes in the two *Corydalis* chloroplast genomes

Group of genes	Gene names	Number of genes
Photosystem I	*psa*A, *psa*B, *psa*C(×2), *psa*I(×2), *psa*J	5 (2)
Photosystem II	*psb*A, *psb*B, *psb*C, *psb*D, *psb*E, *psb*F, *psb*I, *psb*J, *psb*K, *psb*L, *psb*M, *psb*N, *psb*T, *psb*Z	14
Cytochrome b/f complex	*pet*A, *pet*B*, *pet*D*, *pet*G, *pet*L, *pet*N	6
ATP synthase	*atp*A, *atp*B, *atp*E, *atp*F*, *atp*H, *atp*I	6
NADH‐dehydrogenase	*ndh*A*, *ndh*B*(×2), *ndh*C, *ndh*D(×2), *ndh*E(×2), *ndh*F(×2), *ndh*G(×2), *ndh*H, *ndh*I(×2), *ndh*J, *ndh*K,	11 (6)
RubisCO large subunit	*rbc*L	1
DNA dependent RNA polymerase	*rpo*A, *rpo*B, *rpo*C1*, *rpo*C2	4
Small subunit of ribosome	*rps*2, *rps*3, *rps*4, *rps*7(×2*)*, *rps*8, *rps*11, *rps*12*(×2), *rps*14, *rps*15, *rps*16*, *rps*18, *rps*19	12 (2)
Large subunit of ribosome	*rpl*2*(×2), *rpl*14, *rpl*16*, *rpl*20, *rpl*22, *rpl*23(×2), *rpl*32(×2), *rpl*33, *rpl*36	9 (3)
Proteins of unknown function	*ycf*1, *ycf2*(×2), *ycf*3**, *ycf*4, *ycf*15(×2)	5 (2)
Other genes	*ccs*A(×2), *cem*A, *inf*A, *mat*K, *clp*P**	5 (1)
Transfer RNAs	37 tRNAs(*C*. *tomentella*); 38 tRNAs(*C*. *saxicola*)	37/38
Ribosomal RNAs	*rrn*16S(×2), *rrn*23S(×2), *rrn*4.5S(×2), *rrn*5S(×2)	4 (4)

Note

One or two asterisks followed genes indicate the number of contained introns, respectively. (×2) indicates the number of the repeat unit is 2. The numbers in parenthesis at the line of “Number” indicate the total number of repeated genes.

### 3.2 Variation in genome structural

**FIGURE 3:**
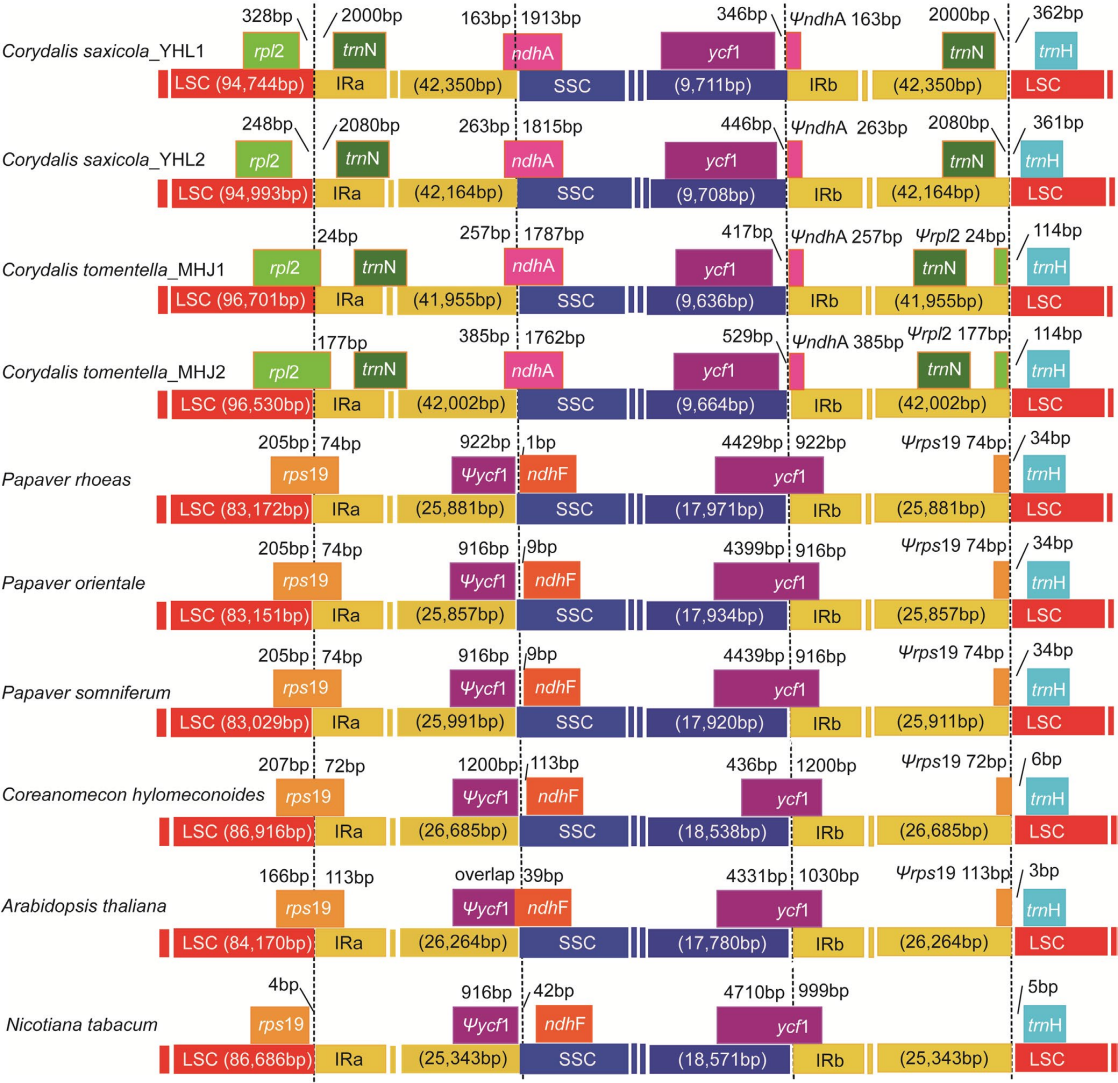
Sequence identity plot comparison of the *C. tomentella* and *C. saxicola* cp genomes. Gray arrows and thick black lines above the alignment indicate genes with their orientation and the position of the inverted repeats (IRs), respectively. A cutoff of 70% identity was used for the plots, and the Y‐scale represents the percent identity ranging from 50% to 100%

VISTA software was used to make multiple comparisons of the *C. tomentella* and *C. saxicola* cp genome sequences, and results show that intraspecific variation was small but there were still some inter‐specific differences (Figure 3). The coding and noncoding regions of *C. saxicola* samples were conserved, while the coding regions of *C. tomentella* samples were conserved but there were differences in several consecutive intergenic regions of *rps1*2*‐clp*P, *clp*P*‐psb*B, and *pet*B*‐psb*H. Comparing *C. tomentella* and *C. saxicola*, the most highly divergent regions mainly were observed in coding regions and intergenic regions, including *rpl*20, *rrn*23s, *trn*H‐GUG, *trn*N‐GUU, *rps*12*‐clp*P, *clp*P*‐psb*B, *pet*B*‐psb*H, and *ycf*1*‐ndh*L. On the basis of morphological features and cluster analysis of DNA barcodes, it was found that the two species are closely related (Ren et al., 2019). The cp genome differences between the two species have potential for use as molecular markers for species authentication.

**FIGURE 4:**
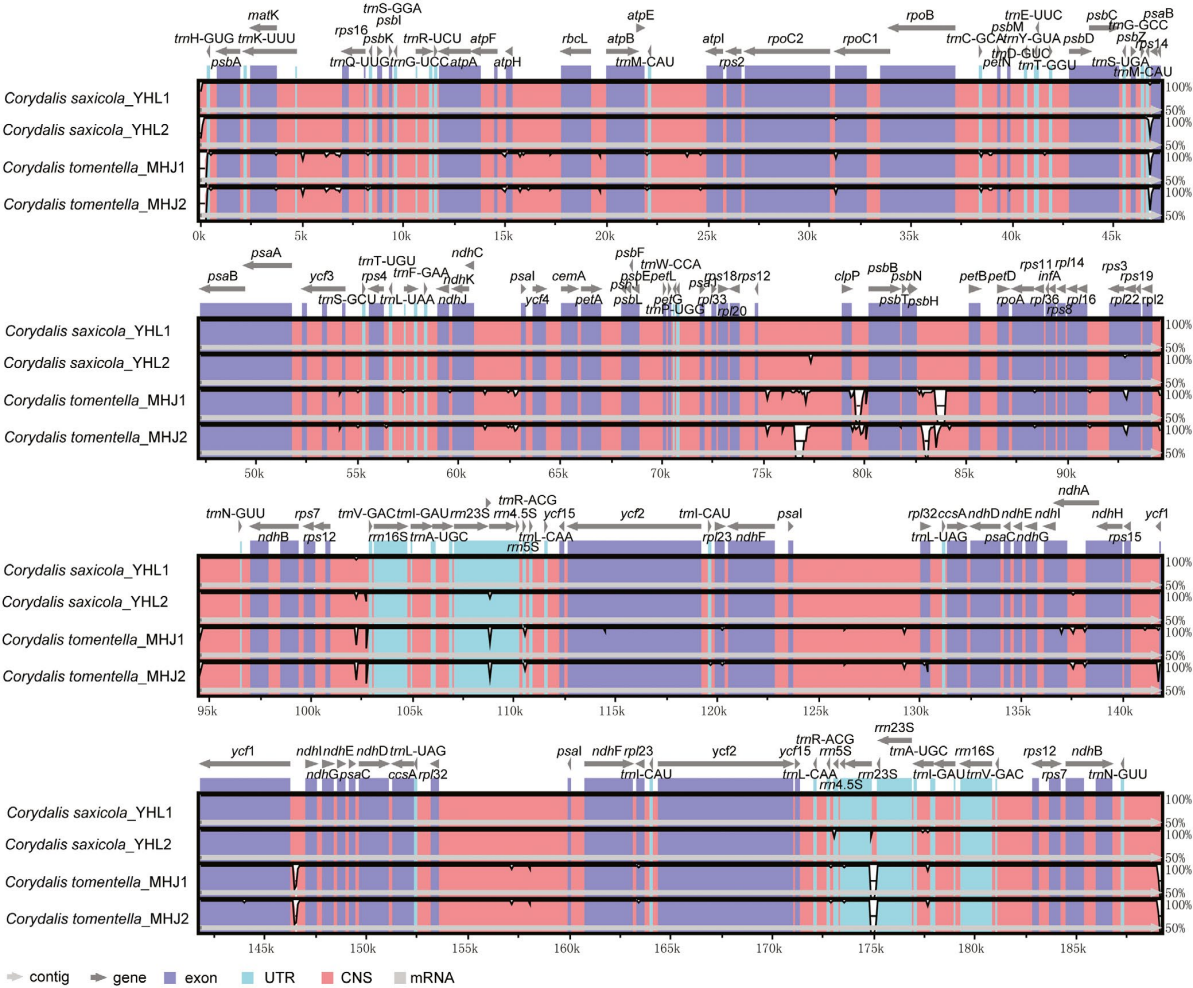
Sequence identity plot comparison of the cp genomes of *C. tomentella*, *C. saxicola*, *P. somniferum*, *P. rhoeas*, and *C. hymenoides*. Gray arrows and thick black lines above the alignment indicate genes with their orientation and the position of the inverted repeats (IRs), respectively. A cutoff of 70% identity was used for the plots, and the Y‐scale represents the percent identity ranging from 50% to 100%

Comparisons with the *N. tabacum* outgroup and Papaveraceae family plants *P. rhoeas*, *P. orientale*, *P. somniferum*, and *C. hylomeconoides* showed that *C. tomentella* and *C. saxicola* cp genomes have distinct cp genome structures. The differences included genome size, number of genes, and a disruption of gene collinearity (Figure 4). First, the *C. tomentella* and *C. saxicola* cp genome sizes (189.1–190.2 kb) were larger than those of *N. tabacum* (155.9 kb) and *P. somniferum* (152.9 kb). Second, the length of intergenic regions in *C. tomentella* and *C. saxicola* cp genomes were longer than those in *N. tabacum* and *P. somniferum*, as seen, for example, in the lengths of intergenic regions for *psa*l/*rpl32* (7 kb) in the IR region and *rps12*/*clp*P (5 kb) in the LSC region. Third, *C. tomentella* and *C. saxicola* cp genome structures were significantly different from those of the other six species, including large‐scale gene replication, movement, reversal, and changes in the number and arrangement of genes. Fourth, *C. tomentella* and *C. saxicola* IR regions *were* highly dilated (41.9–42.5 kb). The *ndh*F, *ndh*D, *ndh*L, *ndh*G, *ndh*E, *psa*C, *ccs*A, *trn*L‐UAG, and *rpl32* genes, usually located in the SSC region, migrated to the IR regions to become double‐copy genes (Figure 1). A few *rpl*19 and *rpl*2 genes migrated from the IR region to the LSC region. In particular, in *C. tomentella* and *C. saxicola*, there is a large fragment (containing *rpl*23, *trn*L‐CAU, *ycf*2, *ycf*15, and *trn*L‐CAA) that moved within the IR region. Gene migration increased the length of the IR region and decreased the length of the SSC region. Fifth, the LSC region was highly conserved, but the *acc*D gene was lost and the position of the *rbc*L gene changed substantially. In short, both the coding and noncoding regions of *C. tomentella* and *C. saxicola* cp genomes differ greatly from those of other Papaveraceae and tobacco.

**FIGURE 5:**
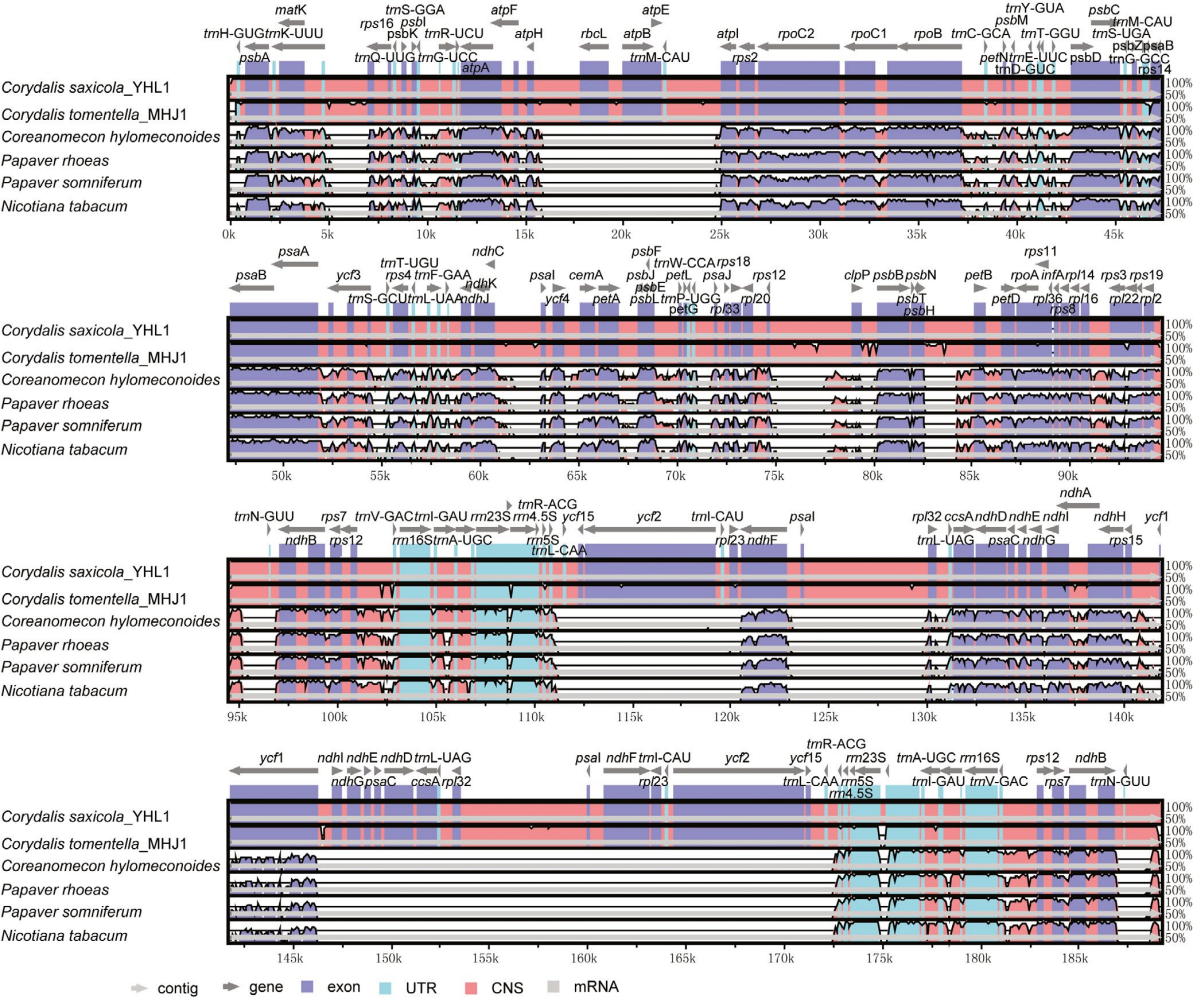
Comparison of the borders of LSC, SSC, and IR regions among the eight chloroplast genomes. Number above the gene features indicates the distance between the ends of genes and the border sites. Ψ: pseudogenes

Inverted repeat regions are the most conserved regions in the plant plastome, contraction, and expansion at their borders are regarded as the major causes of size variation (Chumley et al., 2006; Xin et al., 2019). We selected four phylogenetically close species (*P. rhoeas*, *P. orientale*, *P. somniferum*, and *C. hylomeconoides*) and two model species (*N. tabacum* and *A. thaliana*) as references for cp genome structure comparisons. Figure 5 displays the detailed information about the boundaries between IR/SSC and IR/LSC in the eight species.

Except for *C. tomentella* and *C. saxicola*, the IRb/SSC boundaries were generally positioned in the coding region of the *ycf*1 gene, resulting in duplication of the 3′ end of this gene. This duplication also produced a variably sized pseudogene *ycf*1 at the IRa/SSC border. The length of the *ycf*1 pseudogene varied from 916 to 1,200 bp. However, the *ycf*1 genes in *C. tomentella* and *C. saxicola* cp genomes have been transferred to the SSC region to become a single copy gene. Except for *C. tomentella*, *C. saxicola*, and *N. tabacum*, the LSC/IRb borders of other species were located within the *rps*19 coding region. Correspondingly, a 3′‐truncated *rps*19 pseudogene with a length of 74–113 bp was located at the IRb/LSC border. In the *C. tomentella* cp genome, the LSC/IRb border was located in the *rpl*2 coding region. Additionally, in *C. tomentella* and *C. saxicola* cp genomes, the IRa/SSC boundaries were positioned in the *ndh*A coding region, and *trn*N was situated in the IRa and IRb regions, away from the LSC/IRa and IRb/LSC borders. The *trn*H gene was present in LSC regions, away from the IRb/LSC border.

### 3.3 Codon usage bias, SSRs, and repeat sequences

Coding sequence codon usage patterns for the *C. tomentella* and *C. saxicola* cp genomes were calculated on the basis of relative synonymous codon usage (RSCU) values. We defined codons with RSCU values greater than 1.00 to be used more frequently, and vice versa. All protein‐coding genes in the *C. tomentella* and *C. saxicola* cp genomes were encoded by 52,244 codons and 51,125 codons, respectively (Table S2). The most prevalent amino acid was Leucine in the cp genomes of *C. tomentella* (5,656; 10.83%) and *C. saxicola* (5,528; 10.81%). Conversely, the least frequently utilized amino acid was Cysteine in the cp genomes of these two species (591–634; 1.16%–1.18%). The third position nucleotides in each codon of all the coding genes had a high AT content, at 65.83% and 65.91% for *C. tomentella* and *C. saxicola*, respectively.

Simple sequence repeats are short tandem repeats of 1–6 bp DNA sequences that are widely distributed throughout the cp genome (Lee et al., 2019). In this study, CPGAVAS2 software was used to analyze the sequences and the classification statistics of SSRs with a length greater than or equal to 8 bp. Here, we analyzed the distribution and the type of SSRs contained in *C. tomentella* and *C. saxicola* cp genomes. A total of 172 SSRs were identified in the whole *C. tomentella* cp genome (take MHJ1 as an example), including 100 mono‐, 34 di‐, and one compound nucleotide SSRs. Among all SSR types, A and T were the most commonly used bases and 116 SSRs in the *C. tomentella* cp genome had A, T, or AT repeat units (Table 3 and Table S3). For *C. saxicola*, 170 SSRs (take YHL2 as an example) were categorized as 96 mono‐, 36 di‐, six tri‐ and six compound nucleotide SSRs, including 115 SSRs with A, T, or AT repeat units (Table 3 and Table S3).

**TABLE 3:** Interspersed repeat sequences and tandem repeat sequences of *C. saxicola* and *C. tomentella*

Species	Voucher No.	SSR		Interspersed repeat sequences			
		Total	Mono SSR	Total	T	F	P
*Corydalis tomentella*	MHJ1	172	100	111	61	39	11
	MHJ2	174	102	112	62	39	11
*Corydalis saxicola*	YHL1	171	96	132	82	23	27
	YHL2	170	96	133	83	26	24

Abbreviations: F, Forward repeats; P, palindromic repeats; T, tandem repeats.

In addition to SSRs, forward repeats (F) and palindromic repeats (P) are also called interspersed repeat sequences (length ≥ 30 bp). In the *C. tomentella* cp genome, there were 112 interspersed repeat sequences, comprised of 64 tandem repeats, 39 forward repeats, and 11 palindromic repeats (Table 3). A total of 132 long repeats were present in *C. saxicola* cp genome, comprised of 82 tandem repeats, 23 forward repeats, and 27 palindromic repeats (Table 3). Comparing the cp genomes of the two species, the *C. saxicola* genome had a greater total number of repeats than the *C. tomentella* cp genome, and the cp genome repeat content in both species was significantly higher than that of most species.

### 3.4 Phylogenetic analysis

**FIGURE 6:**
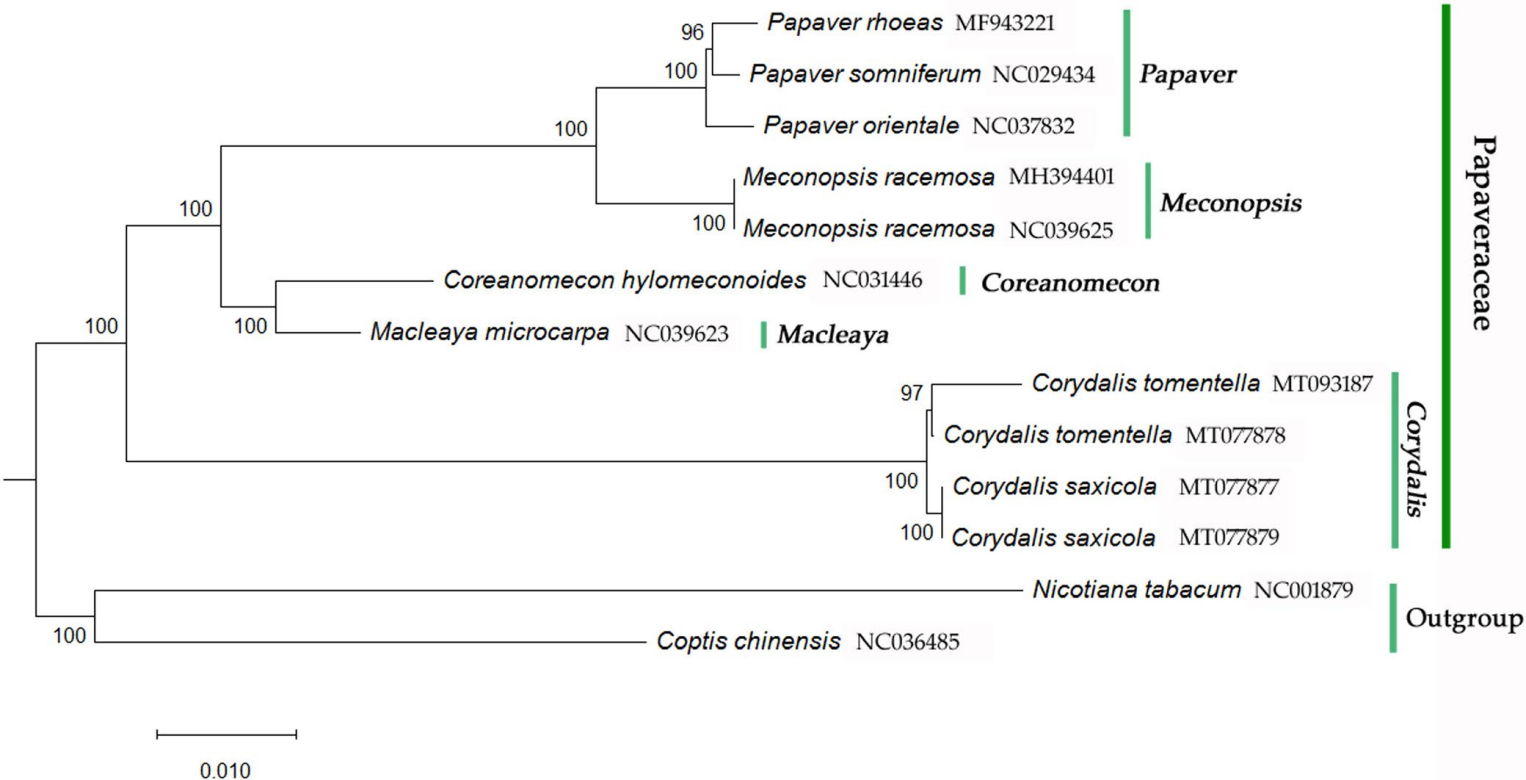
ML tree of *C. saxicola* and *C. tomentella* and its relative species based on common protein coding sequences

With *C. chinensis* and *N. tabacum* as outgroups, 70 common protein coding sequences from 13 cp genome sequences were extracted from *C*. *saxicola*, *C*. *tomentella*, and six Papaveraceae species to build a Maximum Likelihood (ML) phylogenetic tree (Figure 6). The ML tree has high bootstrap values at each node, indicating a highly credible tree. In this ML tree, the Papaveraceae family is monophyletic, and all samples from Papaveraceae are clustered in a clade. In Papaveraceae, the samples from the genus *Papaver* (*P. somniferum*, *P. orientale*, and *P. rhoeas*) are clustered in a clade; the samples from *Corydalis* (*C. saxicola* and *C. tomentella*) are clustered in a clade; the samples from *Meconopsis* (*M. racemosa*) are clustered in a clade; and *C. hymenoides* and *M. microcarpa* are clustered in a clade. Except for *Coreanomecon* and *Macleaya*, which had only one sample, species in the same genus are clustered into one branch, consistent with previous classification of Papaveraceae genera. At the species level, the *C. saxicola* and *C. tomentella* samples are clustered into separate branches, indicating that the cp genome clustering analysis could effectively distinguish them, while these two closely related species were not monophyletic in the phylogenetic analysis based on short sequence DNA barcodes (Ren et al., 2019). At the same time, *C*. *saxicola* and *C*. *tomentella* are clustered in a clade in the ML phylogenetic tree that is distant from other Papaveraceae genera. It shows that *C. saxicola* and *C*. *tomentella*, both from Sect. *Thalictrifoliae* in *Corydalis*, have a close genetic relationship.

## 4 DISCUSSION

### 4.1 High variability of genome size and the expansion of IRs

*Corydalis saxicola* and *C. tomentella* cp genomes are the large cp genomes due to the expansion of IR regions. Most angiosperms cp genomes are highly conserved, typically 115–165 kb in size and possessing a quadripartite structure with two IR regions (IRa and IRb) separating the LSC region and the SSC region (Xin et al., 2019). The sizes of *C. saxicola* and *C. tomentella* cp genomes are larger than those of most flowering plants, such as *N. tabacum* (Sajjad et al., 2016; Shinozaki et al., 1986; Yukawa et al., 2006), 30–40 kb larger than those reported genomes in Papaveriaceae, such as *P. somniferum* (Sun et al., 2016) and *C. hymenoides* (Kim & Kim, 2016). Distinctions between different cp genomes mainly result from the variability of the length and direction of IR regions (Duan et al., 2020). In terms of length, IR regions of the genus *Taxodium* (*T. distichum*, *T. mucronatum* and *T. ascendens*) contracted to about 282 bp (Saski et al., 2005), while IR regions were entirely absent in *Pisum sativum* and *Cryptomeria japonica* (Hirao et al., 2008; Ki & Hae, 2005). In contrast, the length of *Pelargonium hortorum* IR regions expanded to 76 kb (Duan et al., 2020). Numerous studies have shown that IR region lengths are the main factor influencing cp genome size (Yan et al., 2017). In our study, IR region lengths for the two newly sequenced species were 41,955 to 42,350 bp, which significantly increased their cp genome sizes over that of other Papaveraceae species. Genes normally located in the SSC region, such as *ndh*C, *ndh*D, *ndh*E, *ndh*F, *ndh*G, *ndh*L, *rpl*32, and *trn*L‐UAG, have moved to IR regions, contributing to the expanded size of *C. saxicola* and *C. tomentella* IRs.

### 4.2 Gene inversions, duplications, and deletions

Inversions usually serve as useful phylogenetic markers (Cosner et al., 2004; Kim et al., 2005). An up to 9 kb inversion containing five genes (*rpl*23, *ycf*2, *ycf*15, *trn*L‐CAU, and *trn*L‐CAA) was found in the IR regions of *C. tomentella* and *C. saxicola* cp genomes. Relatively large inversions have been found in the cp genomes of some other flowering plants. The 22.8 kb inversion is present in all Asteraceae, except *Barnadesioideae* (Jansen & Palmer, 1987; Martin et al., 2014), the 36 and 78 kb inversions have been detected in core genistoid legumes and Fabaceae subtribe *Phaseolinae*, respectively (Bruneau & Palmer, 1990; Jansen, 2011). These distinctive inversions serve as phylogenetic markers. The inversion in *C. tomentella* and *C. saxicola* is quite distinct from other sequenced Papaveraceae species. To determine if it can be used as a phylogenetic marker of genus *Corydalis*, more species will need to be sequenced. In some plants, the large inversions have been found to be associated with short inverted repeats in cp genome (Joachim et al., 2017; Yi et al., 2013). In Geraniaceae, Campanulaceae and some Fabaceae species, a mass of short inverted repeats have been found to be present at their inversion endpoints (Cosner et al., 2004; Yan et al., 2017). However, we didn't detect large numbers of short inverted repeats emerged in inversion endpoints in *C. tomentella* and *C. saxicola*.

Several NDH (NADH dehydrogenase‐like) genes (*ndh*D, *ndh*E, *ndh*F, *ndh*G, *ndh*L) are duplicated in the *C. tomentella* and *C. saxicola* cp genomes, which could provide an explanation for their robust adaptability to harsh environments. Large‐scale duplication of cp genes tends to occur only in highly rearranged genomes and can be explained by repeated expansion and contraction of IRs (Mercedes & Bartolomé, 2010; Ruhlman et al., 2015). In this study, genes that are normally located in the SSC region (*ndh*D, *ndh*E, *ndh*F, *ndh*G, *ndh*L, *psa*C, *rpl*32, *ccs*A, and *trn*L‐UAG) have migrated to IRs resulting in IR expansion and gene duplication. We found that most of these duplicated genes belong to the NDH complex. Because plastid NDH genes are dispensable under optimal growth conditions, they have been lost in a number of autotrophic and heterotrophic lineages, although they are widely retained across land plants (Ruhlman et al., 2015; Yan et al., 2017). For example, plastid NDH genes have been partially lost or pseudogenized in parasitic plants, such as several orchids and *Petrosavia* (Petrosaviaceae), and autotrophs plants, such as *Najas* (Hydrocharitaceae) and *Erodium* (Geraniaceae) (Mercedes & Bartolomé, 2010), even they have been completely lost in *Selaginella tamariscina* (Xu et al., 2018). Conversely, it is rare for NDH genes to undergo large‐scale duplication and augmentation, and the effects of the increased genes resulting from gene duplication on plant growth and development have rarely been discussed in previous research. The NDH complex participates in photosystem I (PSI) cyclic electron flow (CEF), chlororespiration. NDH‐dependent CEF provides additional pH change and ATP for CO_2_ assimilation and alleviates oxidative stress caused by stromal over‐reduction under stress conditions (Ruhlman et al., 2015). The nonphotochemical quenching ability of NDH deficient mutants decreased under mild drought (Sergi et al., 2005). NDH deficient mutants grow slowly at low humidity (Horvath, 2000). Under strong light, tobacco *ndh*B mutants were more susceptible to photobleaching (Sergi et al., 2005). Under heat stress conditions, NDH‐mediated cyclic and chlororespiratory electron transport are accelerated, mitigating photo‐oxidative damage, and inhibition of CO2 assimilation caused by high temperature (Ju et al., 2003). *Corydalis tomentella* and *C. saxicola* mainly grow in dry cracks of limestone, a unique environment with little available soil and water (Ren et al., 2019) (Figure 1). So they have long been subjected to extreme environmental conditions, such as high temperature, drought, and low light. In view of NDH gene functions in plant defense against various environmental stresses, the doubling of NDH genes those results from IR expansion could lead to overexpression of these doubled genes, which would be helpful for adaptation to harsh environmental conditions. The special structure of the *C. tomentella* and *C. saxicola* cp genomes provides a clue that could explain their robust adaptation to harsh environments.

The *acc*D gene was absent in *C. saxicola* and *C. tomentella* cp genomes. Usually, gene content is highly conserved among photosynthetic angiosperm cp genomes (Jansen et al., 2007; Yan et al., 2017), but in a very few plants, for example, legumes and Circaeasteraceae (Magee et al., 2010; Xu et al., 2018), a number of genes have been lost or pseudogenized. The loss of *acc*D in the cp genome is mirrored in other plant taxa, such as grasses, Circaeasteraceae, and Oleaceae (Joachim et al., 2017; Yan et al., 2017). The *acc*D gene encodes an acetyl‐CoA carboxylase subunit and is an important regulator of carbon flow entering the fatty acid biosynthesis pathway (Rousseau‐Gueutin et al., 2013). It is known to be essential for leaf development in angiosperms (Hong et al., 2017; Kode et al., 2005). Recent research has shown that the *acc*D gene present in the plastome of most angiosperms is functional (Hong et al., 2017; Rousseau‐Gueutin et al., 2013). Furthermore, several studies have shown that the *acc*D gene has been transferred into the nucleus, and the proteins it encodes are transported from the nucleus to the chloroplast to function in the form of a transfer peptide (Joachim et al., 2017; Liu et al., 2016). Whether the *C. tomentella* and *C. saxicola* *acc*D genes have been lost or transferred to the nucleus, the effects on development are currently unknown.

### 4.3 Potential application of cp genome in phylogenetic research of *Corydalis* and Papaveraceae

By exhibiting high species identification power that accurately distinguished two closely related species (*C*. *saxicola* and *C*. *tomentella*), cp genomes have demonstrated a great potential for use as a super‐barcode to discriminate *Corydalis* species. *Corydalis*, is considered to be one of the most taxonomically complex taxa (Wu et al., 1996). It is extremely difficult to depend on morphological characteristics for *Corydalis* species identification. Single‐locus DNA barcodes lack adequate variation in closely related taxa. Researches using short sequence gene fragments and DNA barcodes showed that both nuclear genome (ITS/ITS2) sequence and cp genome (*mat*K/*rbc*L/*rps*16) sequence produced unsatisfactory taxonomic identifications within *Corydalis* (Ren et al., 2019; Wang, 2006). Cp genomes, exhibiting many advantages, including a moderate size and an appropriate frequency of nucleotide substitutions that can provide sufficient mutation sites (Yan et al., 2017), have been successfully used in the identification of various taxa, such as genera *Epimedium* (Guo et al., 2019), *Fritillaria* (Yan et al., 2018), *Epipremnum* (Tian et al., 2018), and *Papaver* (Zhou et al., 2017). In this study, *C*. *tomentella* and *C*. *saxicola*, two closely related species from Sect. *Thalictrifoliae* in *Corydalis*, are clustered into two branches in the phylogenetic tree, which indicates they could be accurately distinguished by cp genome analysis. While, in the phylogenetic analysis based on short sequences of DNA barcodes, these two related species were not monophyletic and couldn't be effectively distinguished. Recent barcoding studies have placed a greater emphasis on the use of whole‐cp genome sequences, which are now more readily available as a consequence of improving sequencing technologies (Li et al., 2015). The demonstrated use of cp genomics in *Corydalis* species identification suggests that it has a great potential for taxonomic identification of this genus.

The cp genome also efficiently identified every genus of Papaveraceae in this study. The evolution rates of coding and noncoding regions are significantly different in cp genomes, enabling cp genome use for systematic analysis of different phylogenetic ranks (Clegg et al., 1994). The genus *Corydalis* belongs to Papaveraceae Fumarioideae (Corydaleae) and the phylogenetic relationships of this genus remain controversial (Wu et al., 1996). Recent studies have tended to treat the genus *Corydalis* as an independent Fumariaceae family because the morphological characteristics of this genus constitute a unique evolutionary series (Pérez‐Gutiérrez et al., 2012; Wu & Lu, 2003; Zhang et al., 2008). In this study, a Papaveraceae phylogenetic tree, built using common protein CDS, shows that every genus is clustered into one separate clade. However, the clade of *Corydalis* is far from the other genera of Papaveraceae. Combined with the substantial differences in cp genome structures between *Corydalis* and the other Papaveraceae genera, it will be necessary to analyze more representative species to reveal the phylogenetic relationship of *Corydalis*.

## CONFLICT OF INTEREST

None declared.

## AUTHOR CONTRIBUTION

**Fengming Ren:** Conceptualization (equal); Writing‐original draft (equal). **Liqiang Wang:** Data curation (equal); Software (equal); Writing‐review & editing (equal). **Ying Li:** Formal analysis (equal); Software (equal). **Wei Zhuo:** Formal analysis (equal); Writing‐original draft (equal). **Zhichao Xu:** Formal analysis (equal); Software (equal). **Haojie Guo:** Software (equal). **Yan Liu:** Writing‐review & editing (equal). **Ranran Gao:** Validation (equal). **Jingyuan Song:** Conceptualization (equal); Funding acquisition (equal); Writing‐review & editing (equal).

## Supporting information

Figure S1

Table S1

Table S2

Table S3

## Data Availability

The DNA sequences reported in this study have been deposited in the National Center for Biotechnology Information (NCBI) genome database, and Genbank accessions: MT093187, MT07787‐MT07789. All sequences used in phylogenetic analysis of Papaveraceae are available from NCBI (Accession numbers: see the “Phylogenetic analysis of Papaveraceae” in Section 3).
