# Supplementary material for: Highly variable chloroplast genome from two endangered Papaveraceae lithophytes Corydalis tomentella and Corydalis saxicola
Source: Ecol Evol. 2021 Mar 19;11(9):4158–71. doi: 10.1002/ece3.7312 (PMC8093665; doi:10.1002/ece3.7312)
Supplement: Supplementary file 1 — Figure S1 [file ECE3-11-4158-s003.docx]

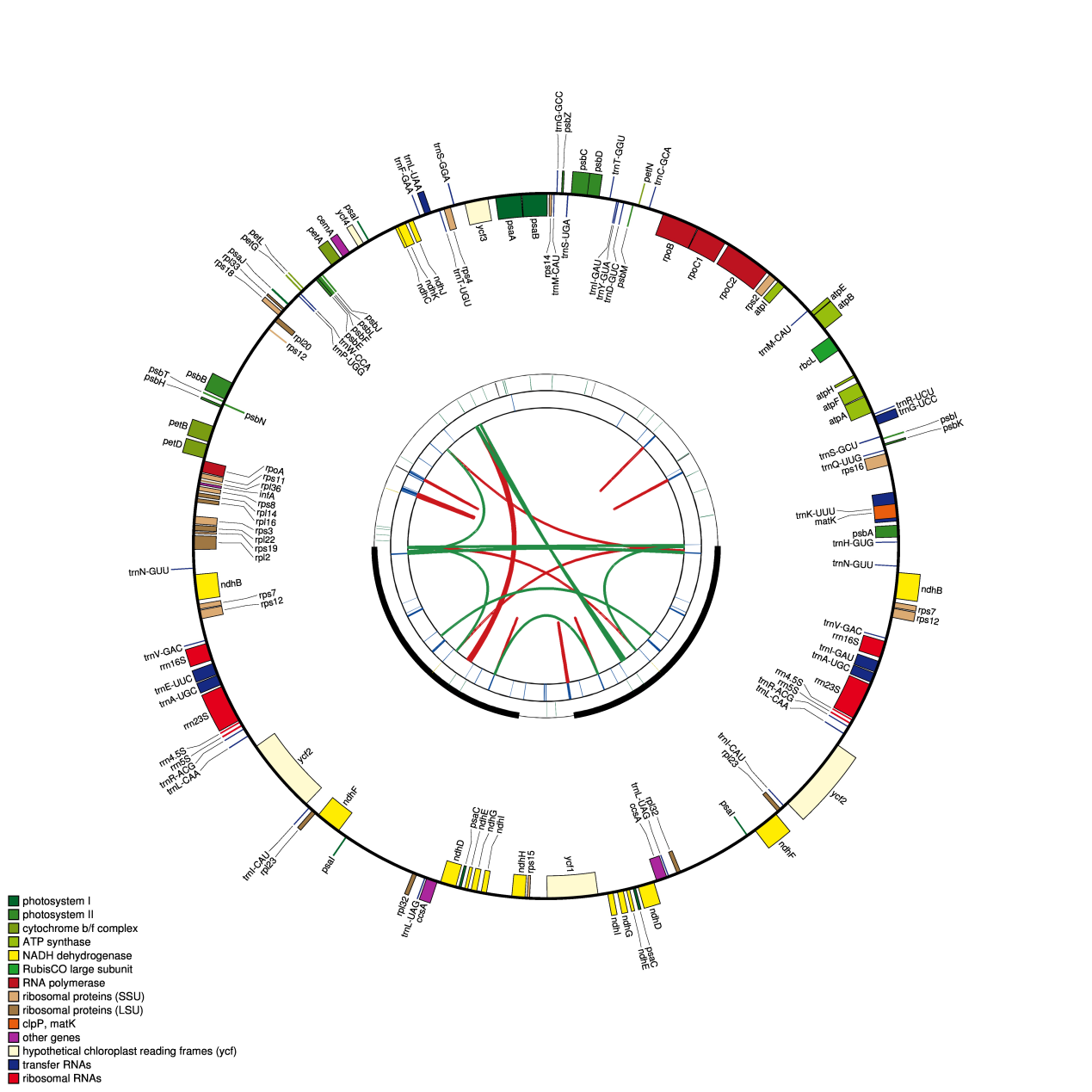


**Figure S1.** Schematic representation of the chloroplast genomes of *C. saxicola*. The map contains four rings. From the center going outward, the first circle shows forward and reverse repeats connected with red and green arcs, respectively. The next circle shows tandem repeats marked with short bars. The third circle shows microsatellite sequences identified by MISA. The fourth circle is drawn using drawgenemap and shows the gene structure of the plastome. The genes are colored on the basis of their functional categories. Genes inside and outside of the circle are transcribed in clockwise and counterclockwise directions, respectively. LSC: large single copy; SSC: small single copy; IR: inverted repeat.
