# Supplementary material for: Highly variable chloroplast genome from two endangered Papaveraceae lithophytes Corydalis tomentella and Corydalis saxicola
Source: Ecol Evol. 2021 Mar 19;11(9):4158–71. doi: 10.1002/ece3.7312 (PMC8093665; doi:10.1002/ece3.7312)
Supplement: Supplementary file 2 — Table S1 [file ECE3-11-4158-s001.docx]

**Table S1.** Lengths of introns and exons of the *C. tomentella* and *C. saxicola* broken genes

**Table S1-1.** Lengths of introns and exons of the *C. tomentella* MHJ1 broken genes

| **Gene** | **Strand** | **Start** | **End** | **ExonI** | **IntronI** | **ExonII** | **IntronII** | **ExonIII** |
| --- | --- | --- | --- | --- | --- | --- | --- | --- |
| *trn*K-UUU | - | 1919 | 4468 | 37 | 2478 | 35 |  |  |
| *rps*16 | - | 6738 | 7823 | 41 | 832 | 213 |  |  |
| *trn*S-CGA | + | 10349 | 11105 | 31 | 664 | 62 |  |  |
| *atp*F | - | 13074 | 14335 | 145 | 707 | 410 |  |  |
| *trn*C-ACA | + | 22038 | 22701 | 39 | 569 | 56 |  |  |
| *rpo*C1 | - | 30843 | 33616 | 432 | 746 | 1596 |  |  |
| *ycf*3 | - | 52846 | 54894 | 124 | 771 | 230 | 765 | 159 |
| *trn*L-UAA | + | 57845 | 58412 | 35 | 483 | 50 |  |  |
| *rps*18 | + | 73289 | 73717 | 36 | 60 | 333 |  |  |
| *rpl*20 | - | 73871 | 74326 | 279 | 30 | 147 |  |  |
| *rps*3 | - | 93979 | 94668 | 162 | 30 | 498 |  |  |
| *rpl*2 | - | 95609 | 96998 | 295 | 619 | 476 |  |  |
| *ndh*B | - | 98945 | 101185 | 775 | 708 | 758 |  |  |
| *trn*E-UUC | + | 106716 | 107731 | 32 | 944 | 40 |  |  |
| *trn*A-UGC | + | 107796 | 108668 | 37 | 800 | 36 |  |  |
| *trn*A-UGC | - | 178281 | 179153 | 37 | 800 | 36 |  |  |
| *trn*E-UUC | - | 179218 | 180233 | 32 | 944 | 40 |  |  |
| *ndh*B | + | 185764 | 188004 | 775 | 708 | 758 |  |  |
| *ndh*A | - | 138612 | 140443 | 552 | 962 | 318 |  |  |

**Table S1-2.** Lengths of introns and exons of the *C. tomentella* MHJ2 broken genes

| **Gene** | **Strand** | **Start** | **End** | **ExonI** | **IntronI** | **ExonII** | **IntronII** | **ExonIII** |
| --- | --- | --- | --- | --- | --- | --- | --- | --- |
| *trn*K-UUU | - | 1919 | 4468 | 37 | 2478 | 35 |  |  |
| *rps*16 | - | 6739 | 7819 | 41 | 827 | 213 |  |  |
| *trn*S-CGA | + | 10345 | 11101 | 31 | 664 | 62 |  |  |
| *atp*F | - | 13070 | 14330 | 145 | 706 | 410 |  |  |
| *trn*C-ACA | + | 22038 | 22701 | 39 | 569 | 56 |  |  |
| *rpo*C1 | - | 30853 | 33626 | 432 | 746 | 1596 |  |  |
| *ycf*3 | - | 52849 | 54902 | 124 | 776 | 230 | 765 | 159 |
| *trn*L-UAA | + | 57857 | 58424 | 35 | 483 | 50 |  |  |
| *rps*18 | + | 73307 | 73735 | 36 | 60 | 333 |  |  |
| *rpl*20 | - | 73889 | 74344 | 279 | 30 | 147 |  |  |
| *rps*3 | - | 93809 | 94498 | 162 | 30 | 498 |  |  |
| *rpl*2 | - | 95439 | 96828 | 295 | 619 | 476 |  |  |
| *ndh*B | - | 98775 | 101015 | 775 | 708 | 758 |  |  |
| *trn*E-UUC | + | 106551 | 107566 | 32 | 944 | 40 |  |  |
| *trn*A-UGC | + | 107631 | 108503 | 37 | 800 | 36 |  |  |
| *ndh*A | - | 138267 | 140314 | 553 | 962 | 533 |  |  |
| *trnA-UGC* | - | 178227 | 179099 | 37 | 800 | 36 |  |  |
| *trn*E-UUC | - | 179164 | 180179 | 32 | 944 | 40 |  |  |
| *ndh*B | + | 185715 | 187955 | 775 | 708 | 758 |  |  |

**Table S1-3.** Lengths of introns and exons of the *C. saxicola* YHL1 broken genes

| **Gene** | **Strand** | **Start** | **End** | **ExonI** | **IntronI** | **ExonII** | **IntronII** | **ExonIII** |
| --- | --- | --- | --- | --- | --- | --- | --- | --- |
| *trn*K-UUU | - | 2107 | 4656 | 37 | 2478 | 35 |  |  |
| *rps*16 | - | 6943 | 8023 | 41 | 827 | 213 |  |  |
| *trn*S-CGA | + | 10552 | 11308 | 31 | 664 | 62 |  |  |
| *atp*F | - | 13277 | 14537 | 145 | 706 | 410 |  |  |
| *trn*C-ACA | + | 22254 | 22916 | 39 | 568 | 56 |  |  |
| *rpo*C1 | - | 31200 | 33855 | 432 | 745 | 1479 |  |  |
| *ycf*3 | - | 52263 | 54316 | 124 | 776 | 230 | 765 | 159 |
| *trn*L-UAA | + | 57268 | 57835 | 35 | 483 | 50 |  |  |
| *rps*18 | + | 72722 | 73156 | 36 | 60 | 339 |  |  |
| *rpl*20 | - | 73310 | 73765 | 279 | 30 | 147 |  |  |
| *rps*3 | - | 92013 | 92702 | 162 | 30 | 498 |  |  |
| *rpl*2 | - | 93675 | 95050 | 292 | 608 | 476 |  |  |
| *ndh*B | - | 96999 | 99239 | 775 | 708 | 758 |  |  |
| *trn*E-UUC | + | 104770 | 105785 | 32 | 944 | 40 |  |  |
| *trn*A-UGC | + | 105850 | 106725 | 37 | 803 | 36 |  |  |
| *trn*A-UGC | - | 177175 | 178050 | 37 | 803 | 36 |  |  |
| *trn*E-UUC | - | 178115 | 179130 | 32 | 944 | 40 |  |  |
| *ndh*B | + | 184661 | 186901 | 775 | 708 | 758 |  |  |
| *trn*K-UUU | - | 2107 | 4656 | 37 | 2478 | 35 |  |  |

**Table S1-4.** Lengths of introns and exons of the *C. saxicola* YHL2 broken genes

| **Gene** | **Strand** | **Start** | **End** | **ExonI** | **IntronI** | **ExonII** | **IntronII** | **ExonIII** |
| --- | --- | --- | --- | --- | --- | --- | --- | --- |
| *trn*K-UUU | - | 2106 | 4655 | 37 | 2478 | 35 |  |  |
| *rps*16 | - | 6942 | 8022 | 41 | 827 | 213 |  |  |
| *trn*S-CGA | + | 10551 | 11307 | 31 | 664 | 62 |  |  |
| *atp*F | - | 13276 | 14536 | 145 | 706 | 410 |  |  |
| *trn*C-ACA | + | 22253 | 22915 | 39 | 568 | 56 |  |  |
| *rpo*C1 | - | 31062 | 33855 | 432 | 745 | 1617 |  |  |
| *ycf*3 | - | 52329 | 54382 | 124 | 776 | 230 | 765 | 159 |
| *trn*L-UAA | + | 57334 | 57901 | 35 | 483 | 50 |  |  |
| *rps*18 | + | 72788 | 73222 | 36 | 60 | 339 |  |  |
| *rpl*20 | - | 73376 | 73831 | 279 | 30 | 147 |  |  |
| *rps*3 | - | 92238 | 92927 | 162 | 30 | 498 |  |  |
| *rpl*2 | - | 93925 | 95301 | 292 | 609 | 476 |  |  |
| *ndh*B | - | 97250 | 99490 | 775 | 708 | 758 |  |  |
| *trn*E-UUC | + | 105155 | 106170 | 32 | 944 | 40 |  |  |
| *trn*A-UGC | + | 106235 | 107107 | 37 | 800 | 36 |  |  |
| *ndh*A | - | 136894 | 138972 | 553 | 993 | 533 |  |  |
| *trn*A-UGC | - | 176917 | 177789 | 37 | 800 | 36 |  |  |
| *trn*E-UUC | - | 177854 | 178869 | 32 | 944 | 40 |  |  |
| *ndh*B | + | 184534 | 186774 | 775 | 708 | 758 |  |  |
