# Supplementary material for: Highly variable chloroplast genome from two endangered Papaveraceae lithophytes Corydalis tomentella and Corydalis saxicola
Source: Ecol Evol. 2021 Mar 19;11(9):4158–71. doi: 10.1002/ece3.7312 (PMC8093665; doi:10.1002/ece3.7312)
Supplement: Supplementary file 3 — Table S2 [file ECE3-11-4158-s002.docx]

**Table S2.** Codon usage frequency of *C.tomentella* and *C. saxicola*

**Table S2-1**. Codon usage frequency of *C.tomentella* MHJ1

| Codon | Amino acid | Frequency(%) | Number |  | Codon | Amino acid | Frequency(%) | Number |
| --- | --- | --- | --- | --- | --- | --- | --- | --- |
| GCA | A | 14.949 | 781 |  | CCA | P | 12.824 | 670 |
| GCC | A | 8.69 | 454 |  | CCC | P | 8.786 | 459 |
| GCG | A | 7.81 | 408 |  | CCG | P | 6.661 | 348 |
| GCT | A | 21.84 | 1141 |  | CCT | P | 16.117 | 842 |
| TGC | C | 3.273 | 171 |  | CAA | Q | 25.457 | 1330 |
| TGT | C | 8.479 | 443 |  | CAG | Q | 9.436 | 493 |
| GAC | D | 9.57 | 500 |  | AGA | R | 17.208 | 899 |
| GAT | D | 29.247 | 1528 |  | AGG | R | 7.695 | 402 |
| GAA | E | 35.143 | 1836 |  | CGA | R | 14.356 | 750 |
| GAG | E | 15.121 | 790 |  | CGC | R | 3.771 | 197 |
| TTC | F | 23.275 | 1216 |  | CGG | R | 5.819 | 304 |
| TTT | F | 36.636 | 1914 |  | CGT | R | 12.748 | 666 |
| GGA | G | 23.792 | 1243 |  | AGC | S | 5.895 | 308 |
| GGC | G | 9.207 | 481 |  | AGT | S | 15.217 | 795 |
| GGG | G | 13.935 | 728 |  | TCA | S | 15.389 | 804 |
| GGT | G | 19.122 | 999 |  | TCC | S | 14.7 | 768 |
| CAC | H | 6.508 | 340 |  | TCG | S | 9.417 | 492 |
| CAT | H | 18.816 | 983 |  | TCT | S | 21.495 | 1123 |
| ATA | I | 22.931 | 1198 |  | ACA | T | 15.466 | 808 |
| ATC | I | 17.476 | 913 |  | ACC | T | 10.374 | 542 |
| ATT | I | 37.746 | 1972 |  | ACG | T | 6.852 | 358 |
| AAA | K | 35.794 | 1870 |  | ACT | T | 19.715 | 1030 |
| AAG | K | 15.542 | 812 |  | GTA | V | 17.303 | 904 |
| CTA | L | 14.739 | 770 |  | GTC | V | 7.695 | 402 |
| CTC | L | 9.379 | 490 |  | GTG | V | 8.652 | 452 |
| CTG | L | 9.303 | 486 |  | GTT | V | 18.777 | 981 |
| CTT | L | 22.548 | 1178 |  | TGG | W | 18.95 | 990 |
| TTA | L | 28.922 | 1511 |  | TAC | Y | 6.317 | 330 |
| TTG | L | 23.371 | 1221 |  | TAT | Y | 28.233 | 1475 |
| ATG | M | 24.922 | 1302 |  | TAA | * | 2.163 | 113 |
| AAC | N | 11.542 | 603 |  | TAG | * | 1.684 | 88 |
| AAT | N | 33.286 | 1739 |  | TGA | * | 1.914 | 100 |

**Table S2-2** Codon usage frequency of *C.tomentella* MHJ2

| Codon | Amino acid | Frequency(%) | Number |  | Codon | Amino acid | Frequency(%) | Number |
| --- | --- | --- | --- | --- | --- | --- | --- | --- |
| GCA | A | 14.745 | 777 |  | CCA | P | 12.658 | 667 |
| GCC | A | 8.635 | 455 |  | CCC | P | 8.824 | 465 |
| GCG | A | 7.591 | 400 |  | CCG | P | 6.699 | 353 |
| GCT | A | 21.406 | 1128 |  | CCT | P | 15.941 | 840 |
| TGC | C | 3.663 | 193 |  | CAA | Q | 25.999 | 1370 |
| TGT | C | 8.369 | 441 |  | CAG | Q | 9.47 | 499 |
| GAC | D | 9.811 | 517 |  | AGA | R | 17.383 | 916 |
| GAT | D | 29.187 | 1538 |  | AGG | R | 8.046 | 424 |
| GAA | E | 35.184 | 1854 |  | CGA | R | 14.518 | 765 |
| GAG | E | 15.732 | 829 |  | CGC | R | 3.833 | 202 |
| TTC | F | 23.532 | 1240 |  | CGG | R | 6.262 | 330 |
| TTT | F | 36.284 | 1912 |  | CGT | R | 12.829 | 676 |
| GGA | G | 23.532 | 1240 |  | AGC | S | 5.75 | 303 |
| GGC | G | 8.786 | 463 |  | AGT | S | 15.125 | 797 |
| GGG | G | 13.645 | 719 |  | TCA | S | 15.599 | 822 |
| GGT | G | 18.882 | 995 |  | TCC | S | 14.593 | 769 |
| CAC | H | 6.566 | 346 |  | TCG | S | 9.261 | 488 |
| CAT | H | 18.56 | 978 |  | TCT | S | 21.141 | 1114 |
| ATA | I | 22.621 | 1192 |  | ACA | T | 15.334 | 808 |
| ATC | I | 17.478 | 921 |  | ACC | T | 10.153 | 535 |
| ATT | I | 37.878 | 1996 |  | ACG | T | 6.509 | 343 |
| AAA | K | 36 | 1897 |  | ACT | T | 19.319 | 1018 |
| AAG | K | 15.334 | 808 |  | GTA | V | 18.066 | 952 |
| CTA | L | 14.992 | 790 |  | GTC | V | 7.932 | 418 |
| CTC | L | 9.261 | 488 |  | GTG | V | 8.711 | 459 |
| CTG | L | 9.147 | 482 |  | GTT | V | 18.863 | 994 |
| CTT | L | 23.076 | 1216 |  | TGG | W | 19.015 | 1002 |
| TTA | L | 28.902 | 1523 |  | TAC | Y | 7.268 | 383 |
| TTG | L | 23.076 | 1216 |  | TAT | Y | 27.839 | 1467 |
| ATG | M | 24.841 | 1309 |  | TAA | * | 2.277 | 120 |
| AAC | N | 11.443 | 603 |  | TAG | * | 1.746 | 92 |
| AAT | N | 32.736 | 1725 |  | TGA | * | 2.144 | 113 |

**Table S2-3.** Codon usage frequency of *C. saxicola* YHL1

| Codon | Amino acid | Frequency(%) | Number |  | Codon | Amino acid | Frequency(%) | Number |
| --- | --- | --- | --- | --- | --- | --- | --- | --- |
| GCA | A | 15.413 | 788 |  | CCA | P | 12.44 | 636 |
| GCC | A | 8.743 | 447 |  | CCC | P | 9.017 | 461 |
| GCG | A | 7.648 | 391 |  | CCG | P | 6.65 | 340 |
| GCT | A | 21.809 | 1115 |  | CCT | P | 15.844 | 810 |
| TGC | C | 3.208 | 164 |  | CAA | Q | 25.545 | 1306 |
| TGT | C | 8.352 | 427 |  | CAG | Q | 9.584 | 490 |
| GAC | D | 9.506 | 486 |  | AGA | R | 17.056 | 872 |
| GAT | D | 29.633 | 1515 |  | AGG | R | 7.315 | 374 |
| GAA | E | 34.719 | 1775 |  | CGA | R | 14.181 | 725 |
| GAG | E | 15.296 | 782 |  | CGC | R | 3.834 | 196 |
| TTC | F | 23.726 | 1213 |  | CGG | R | 5.848 | 299 |
| TTT | F | 36.44 | 1863 |  | CGT | R | 12.831 | 656 |
| GGA | G | 24.489 | 1252 |  | AGC | S | 5.907 | 302 |
| GGC | G | 9.115 | 466 |  | AGT | S | 15.081 | 771 |
| GGG | G | 13.888 | 710 |  | TCA | S | 15.452 | 790 |
| GGT | G | 19.54 | 999 |  | TCC | S | 14.748 | 754 |
| CAC | H | 6.474 | 331 |  | TCG | S | 9.389 | 480 |
| CAT | H | 18.934 | 968 |  | TCT | S | 21.477 | 1098 |
| ATA | I | 22.768 | 1164 |  | ACA | T | 15.198 | 777 |
| ATC | I | 17.056 | 872 |  | ACC | T | 10.269 | 525 |
| ATT | I | 38.083 | 1947 |  | ACG | T | 7.061 | 361 |
| AAA | K | 35.169 | 1798 |  | ACT | T | 19.716 | 1008 |
| AAG | K | 15.002 | 767 |  | GTA | V | 17.526 | 896 |
| CTA | L | 14.729 | 753 |  | GTC | V | 7.785 | 398 |
| CTC | L | 9.369 | 479 |  | GTG | V | 8.88 | 454 |
| CTG | L | 8.978 | 459 |  | GTT | V | 19.032 | 973 |
| CTT | L | 22.866 | 1169 |  | TGG | W | 18.778 | 960 |
| TTA | L | 29.086 | 1487 |  | TAC | Y | 6.416 | 328 |
| TTG | L | 23.1 | 1181 |  | TAT | Y | 28.401 | 1452 |
| ATG | M | 25.115 | 1284 |  | TAA | * | 2.269 | 116 |
| AAC | N | 11.56 | 591 |  | TAG | * | 1.623 | 83 |
| AAT | N | 32.978 | 1686 |  | TGA | * | 2.054 | 105 |

| Codon | Amino acid | Frequency (%) | Number |  | Codon | Amino acid | Frequency (%) | Number |
| --- | --- | --- | --- | --- | --- | --- | --- | --- |
| GCA | A | 15.124 | 786 |  | CCA | P | 12.642 | 657 |
| GCC | A | 8.64 | 449 |  | CCC | P | 8.871 | 461 |
| GCG | A | 7.562 | 393 |  | CCG | P | 6.658 | 346 |
| GCT | A | 21.628 | 1124 |  | CCT | P | 16.24 | 844 |
| TGC | C | 3.29 | 171 |  | CAA | Q | 25.746 | 1338 |
| TGT | C | 8.409 | 437 |  | CAG | Q | 9.486 | 493 |
| GAC | D | 9.429 | 490 |  | AGA | R | 17.106 | 889 |
| GAT | D | 29.594 | 1538 |  | AGG | R | 7.427 | 386 |
| GAA | E | 35.155 | 1827 |  | CGA | R | 14.297 | 743 |
| GAG | E | 15.278 | 794 |  | CGC | R | 3.848 | 200 |
| TTC | F | 23.591 | 1226 |  | CGG | R | 5.753 | 299 |
| TTT | F | 36.54 | 1899 |  | CGT | R | 12.892 | 670 |
| GGA | G | 24.149 | 1255 |  | AGC | S | 5.888 | 306 |
| GGC | G | 9.005 | 468 |  | AGT | S | 15.24 | 792 |
| GGG | G | 13.854 | 720 |  | TCA | S | 15.586 | 810 |
| GGT | G | 19.454 | 1011 |  | TCC | S | 14.547 | 756 |
| CAC | H | 6.504 | 338 |  | TCG | S | 9.198 | 478 |
| CAT | H | 18.742 | 974 |  | TCT | S | 21.32 | 1108 |
| ATA | I | 22.667 | 1178 |  | ACA | T | 15.105 | 785 |
| ATC | I | 17.087 | 888 |  | ACC | T | 10.294 | 535 |
| ATT | I | 38.003 | 1975 |  | ACG | T | 6.985 | 363 |
| AAA | K | 35.867 | 1864 |  | ACT | T | 19.761 | 1027 |
| AAG | K | 15.355 | 798 |  | GTA | V | 17.318 | 900 |
| CTA | L | 14.912 | 775 |  | GTC | V | 7.774 | 404 |
| CTC | L | 9.198 | 478 |  | GTG | V | 8.774 | 456 |
| CTG | L | 9.024 | 469 |  | GTT | V | 18.992 | 987 |
| CTT | L | 22.84 | 1187 |  | TGG | W | 18.645 | 969 |
| TTA | L | 28.901 | 1502 |  | TAC | Y | 6.485 | 337 |
| TTG | L | 22.975 | 1194 |  | TAT | Y | 28.363 | 1474 |
| ATG | M | 25.072 | 1303 |  | TAA | * | 2.213 | 115 |
| AAC | N | 11.718 | 609 |  | TAG | * | 1.636 | 85 |
| AAT | N | 33.365 | 1734 |  | TGA | * | 1.982 | 103 |

**Table S2-4**. Codon usage frequency of C. saxicola YHL2
