# Supplementary material for: Highly variable chloroplast genome from two endangered Papaveraceae lithophytes Corydalis tomentella and Corydalis saxicola
Source: Ecol Evol. 2021 Mar 19;11(9):4158–71. doi: 10.1002/ece3.7312 (PMC8093665; doi:10.1002/ece3.7312)
Supplement: Supplementary file 4 — Table S3 [file ECE3-11-4158-s004.docx]

**Table S3.** Simple repeats (SSR) and their distribution of *C. tomentella* and *C. saxicola*

**Table S3-1.** Simple repeats (SSR) and their distribution of *C. tomentella* MHJ1

| **SSR nr** | **SSR type** | **SSR** | **Start** | **End** |  | **SSR nr** | **SSR type** | **SSR** | **Start** | **End** |
| --- | --- | --- | --- | --- | --- | --- | --- | --- | --- | --- |
| 1 | c | (A)8....(A)9...(CT)4 | 208 | 375 |  | 87 | p1 | (A)8 | 87647 | 87654 |
| 2 | p1 | (A)8 | 3134 | 3141 |  | 88 | p2 | (AT)4 | 91403 | 91410 |
| 3 | p1 | (A)9 | 4044 | 4052 |  | 89 | c | (T)8...(T)9 | 91829 | 91860 |
| 4 | p1 | (T)10 | 5339 | 5348 |  | 90 | p1 | (T)12 | 92996 | 93007 |
| 5 | p1 | (T)8 | 7469 | 7476 |  | 91 | p1 | (T)13 | 93430 | 93442 |
| 6 | p2 | (TA)4 | 8843 | 8850 |  | 92 | p1 | (T)10 | 94489 | 94498 |
| 7 | p1 | (A)8 | 9133 | 9140 |  | 93 | p1 | (T)12 | 95520 | 95531 |
| 8 | p1 | (A)8 | 9549 | 9556 |  | 94 | p3 | (GTA)4 | 97134 | 97145 |
| 9 | p1 | (T)9 | 10173 | 10181 |  | 95 | p1 | (T)8 | 97422 | 97429 |
| 10 | p1 | (T)8 | 10922 | 10929 |  | 96 | p1 | (T)8 | 98888 | 98895 |
| 11 | p1 | (T)11 | 13602 | 13612 |  | 97 | p2 | (CT)4 | 110614 | 110621 |
| 12 | p1 | (T)8 | 13748 | 13755 |  | 98 | p2 | (AG)4 | 112392 | 112399 |
| 13 | p1 | (A)8 | 14204 | 14211 |  | 99 | p1 | (T)8 | 115270 | 115277 |
| 14 | p3 | (ATT)4 | 14484 | 14495 |  | 100 | p2 | (GA)4 | 115907 | 115914 |
| 15 | p1 | (A)8 | 14682 | 14689 |  | 101 | p1 | (T)9 | 117949 | 117957 |
| 16 | p1 | (A)9 | 14792 | 14800 |  | 102 | p3 | (GAT)4 | 118893 | 118904 |
| 17 | c | (AT)9...(A)11 | 17001 | 17069 |  | 103 | p2 | (TC)4 | 120297 | 120304 |
| 18 | p1 | (T)9 | 17174 | 17182 |  | 104 | p2 | (TC)4 | 121143 | 121150 |
| 19 | p2 | (TA)5 | 17384 | 17393 |  | 105 | p2 | (AT)6 | 121415 | 121426 |
| 20 | p2 | (TC)4 | 18457 | 18464 |  | 106 | p1 | (T)10 | 121897 | 121906 |
| 21 | c | (T)9...(A)8 | 19657 | 19762 |  | 107 | p1 | (C)8 | 122336 | 122343 |
| 22 | c | (A)8...(ATA)4 | 22593 | 22649 |  | 108 | p1 | (T)9 | 123095 | 123103 |
| 23 | c | (A)9...(T)8 | 24324 | 24411 |  | 109 | p1 | (A)8 | 124053 | 124060 |
| 24 | p1 | (T)8 | 24517 | 24524 |  | 110 | p2 | (AT)4 | 125128 | 125135 |
| 25 | c* | (TC)4(T)10* | 25534 | 25551 |  | 111 | p4 | (TTCT)3 | 125324 | 125335 |
| 26 | p1 | (T)11 | 26367 | 26377 |  | 112 | c | (A)9...(AT)4 | 125626 | 125670 |
| 27 | p2 | (GC)4 | 26927 | 26934 |  | 113 | p1 | (A)8 | 127141 | 127148 |
| 28 | p1 | (T)10 | 28436 | 28445 |  | 114 | p1 | (T)10 | 127276 | 127285 |
| 29 | p1 | (T)8 | 28547 | 28554 |  | 115 | p2 | (CA)4 | 128663 | 128670 |
| 30 | p2 | (TC)5 | 28907 | 28916 |  | 116 | p2 | (TA)5 | 129496 | 129505 |
| 31 | p1 | (A)8 | 32334 | 32341 |  | 117 | p4 | (CTTT)3 | 129934 | 129945 |
| 32 | p3 | (CAA)4 | 38165 | 38176 |  | 118 | p1 | (A)8 | 130356 | 130363 |
| 33 | p1 | (A)8 | 39243 | 39250 |  | 119 | p1 | (A)8 | 132082 | 132089 |
| 34 | p1 | (T)15 | 39586 | 39600 |  | 120 | c | (TA)4...(T)9 | 132503 | 132555 |
| 35 | p1 | (T)8 | 40571 | 40578 |  | 121 | p1 | (T)8 | 133029 | 133036 |
| 36 | p1 | (A)8 | 41670 | 41677 |  | 122 | p1 | (T)9 | 134168 | 134176 |
| 37 | p1 | (G)8 | 44078 | 44085 |  | 123 | p1 | (A)8 | 135330 | 135337 |
| 38 | c | (AT)5...(T)8 | 44972 | 45035 |  | 124 | p2 | (AG)4 | 135630 | 135637 |
| 39 | p2 | (GA)4 | 45148 | 45155 |  | 125 | p1 | (A)8 | 135828 | 135835 |
| 40 | p1 | (A)12 | 45913 | 45924 |  | 126 | p2 | (TA)5 | 137618 | 137627 |
| 41 | p1 | (C)8 | 46236 | 46243 |  | 127 | p1 | (T)9 | 138382 | 138390 |
| 42 | p1 | (C)9 | 46935 | 46943 |  | 128 | p1 | (A)9 | 139340 | 139348 |
| 43 | p2 | (TC)4 | 47867 | 47874 |  | 129 | p1 | (A)8 | 142233 | 142240 |
| 44 | p1 | (C)8 | 49338 | 49345 |  | 130 | c | (T)8...(T)10 | 143469 | 143533 |
| 45 | p1 | (C)14 | 50698 | 50711 |  | 131 | p1 | (A)8 | 143921 | 143928 |
| 46 | p1 | (A)8 | 52828 | 52835 |  | 132 | p1 | (A)9 | 144357 | 144365 |
| 47 | p1 | (A)8 | 54120 | 54127 |  | 133 | p1 | (T)8 | 144638 | 144645 |
| 48 | p1 | (A)13 | 54997 | 55009 |  | 134 | p1 | (T)13 | 145146 | 145158 |
| 49 | p1 | (T)11 | 55256 | 55266 |  | 135 | p1 | (A)8 | 146855 | 146862 |
| 50 | c | (T)13...(TCTT)3 | 55576 | 55632 |  | 136 | p5 | (TTTTC)3 | 146993 | 147007 |
| 51 | c | (TA)4...(T)11...(T)9 | 56890 | 57005 |  | 137 | p1 | (T)9 | 147149 | 147157 |
| 52 | p1 | (A)9 | 57481 | 57489 |  | 138 | c | (TA)4...(AT)4 | 148023 | 148051 |
| 53 | p1 | (A)9 | 58423 | 58431 |  | 139 | p1 | (A)9 | 148559 | 148567 |
| 54 | p1 | (T)10 | 59392 | 59401 |  | 140 | p2 | (TA)5 | 149322 | 149331 |
| 55 | p1 | (T)8 | 59990 | 59997 |  | 141 | p1 | (T)8 | 151114 | 151121 |
| 56 | c* | (C)8(A)9* | 61780 | 61796 |  | 142 | p2 | (CT)4 | 151312 | 151319 |
| 57 | p1 | (T)10 | 62143 | 62152 |  | 143 | p1 | (T)8 | 151612 | 151619 |
| 58 | p3 | (GGT)4 | 62498 | 62509 |  | 144 | p1 | (A)9 | 152773 | 152781 |
| 59 | p4 | (TTCT)3 | 63701 | 63712 |  | 145 | p1 | (A)8 | 153913 | 153920 |
| 60 | p1 | (T)11 | 64339 | 64349 |  | 146 | c | (A)9...(TA)4 | 154394 | 154446 |
| 61 | p1 | (A)8 | 66945 | 66952 |  | 147 | p1 | (T)8 | 154860 | 154867 |
| 62 | p1 | (T)13 | 67570 | 67582 |  | 148 | p1 | (T)8 | 156586 | 156593 |
| 63 | p1 | (G)8 | 68034 | 68041 |  | 149 | p4 | (AAAG)3 | 157004 | 157015 |
| 64 | c | (AT)4...(A)9 | 68273 | 68338 |  | 150 | p2 | (AT)5 | 157443 | 157452 |
| 65 | p1 | (T)8 | 70374 | 70381 |  | 151 | p2 | (TG)4 | 158279 | 158286 |
| 66 | c | (A)8...(CT)4 | 70687 | 70802 |  | 152 | p1 | (A)10 | 159664 | 159673 |
| 67 | p1 | (A)8 | 72108 | 72115 |  | 153 | p1 | (T)8 | 159801 | 159808 |
| 68 | p2 | (CT)5 | 72595 | 72604 |  | 154 | c | (TA)...(T)9 | 161278 | 161323 |
| 69 | p1 | (T)8 | 72879 | 72886 |  | 155 | p4 | (AAGA)3 | 161613 | 161624 |
| 70 | p1 | (A)9 | 73476 | 73484 |  | 156 | p2 | (AT)4 | 161814 | 161821 |
| 71 | p1 | (A)8 | 73660 | 73667 |  | 157 | p1 | (T)8 | 162889 | 162896 |
| 72 | p1 | (T)9 | 73832 | 73840 |  | 158 | p1 | (A)9 | 163846 | 163854 |
| 73 | p1 | (A)9 | 74433 | 74441 |  | 159 | p1 | (G)8 | 164606 | 164613 |
| 74 | p1 | (T)9 | 74949 | 74957 |  | 160 | p1 | (A)10 | 165043 | 165052 |
| 75 | p3 | (TTG)4 | 75570 | 75581 |  | 161 | p2 | (AT)6 | 165523 | 165534 |
| 76 | c | (AT)8...(AT)4...(GA)4 | 77349 | 77532 |  | 162 | p2 | (GA)4 | 165799 | 165806 |
| 77 | c | (AT)4...(GA)4 | 78226 | 78306 |  | 163 | p2 | (GA)4 | 166645 | 166652 |
| 78 | c | (AT)8...(AT)4...(GA)4 | 78903 | 79086 |  | 164 | p3 | (ATC)4 | 168045 | 168056 |
| 79 | c | (AG)4...(T)9 | 79588 | 79662 |  | 165 | p1 | (A)9 | 168992 | 169000 |
| 80 | p1 | (T)10 | 79875 | 79884 |  | 166 | p2 | (CT)4 | 171034 | 171041 |
| 81 | p2 | (AT)4 | 81381 | 81388 |  | 167 | p1 | (A)8 | 171672 | 171679 |
| 82 | c | (AT)5...(TA)5 | 81619 | 81682 |  | 168 | p2 | (TC)4 | 174549 | 174556 |
| 83 | p2 | (AT)5 | 85562 | 85571 |  | 169 | p2 | (AG)4 | 176328 | 176335 |
| 84 | p1 | (T)8 | 85888 | 85895 |  | 170 | p1 | (A)8 | 188054 | 188061 |
| 85 | p2 | (GA)4 | 86205 | 86212 |  | 171 | p1 | (A)8 | 189520 | 189527 |
| 86 | p1 | (A)8 | 86579 | 86586 |  | 172 | p3 | (TAC)4 | 189804 | 189815 |

**Table S3-2.** Simple repeats (SSR) and their distribution of *C. tomentella* MHJ2

| **SSR nr** | **SSR type** | **SSR** | **Start** | **End** |  | **SSR nr** | **SSR type** | **SSR** | **Start** | **End** |
| --- | --- | --- | --- | --- | --- | --- | --- | --- | --- | --- |
| 1 | c | (A)8...(A)9...(CT)4 | 208 | 375 |  | 88 | p2 | (AT)4 | 91233 | 91240 |
| 2 | p1 | (A)8 | 3134 | 3141 |  | 89 | c | (T)8...(T)9 | 91659 | 91690 |
| 3 | p1 | (A)10 | 4044 | 4053 |  | 90 | p1 | (T)13 | 92826 | 92838 |
| 4 | p1 | (A)8 | 4578 | 4585 |  | 91 | p1 | (T)12 | 93261 | 93272 |
| 5 | p1 | (T)10 | 5340 | 5349 |  | 92 | p1 | (T)10 | 94319 | 94328 |
| 6 | p1 | (T)8 | 7470 | 7477 |  | 93 | p1 | (T)12 | 95350 | 95361 |
| 7 | p2 | (TA)4 | 8839 | 8846 |  | 94 | p3 | (GTA)4 | 96964 | 96975 |
| 8 | p1 | (A)8 | 9129 | 9136 |  | 95 | p1 | (T)8 | 97252 | 97259 |
| 9 | p1 | (A)8 | 9545 | 9552 |  | 96 | p1 | (T)8 | 98718 | 98725 |
| 10 | p1 | (T)9 | 10169 | 10177 |  | 97 | p2 | (CT)4 | 110449 | 110456 |
| 11 | p1 | (T)10 | 13598 | 13607 |  | 98 | p2 | (AG)4 | 112227 | 112234 |
| 12 | p1 | (T)8 | 13743 | 13750 |  | 99 | p1 | (T)8 | 115105 | 115112 |
| 13 | p1 | (A)8 | 14199 | 14206 |  | 100 | p2 | (GA)4 | 115742 | 115749 |
| 14 | p3 | (ATT)4 | 14478 | 14489 |  | 101 | p1 | (T)9 | 117790 | 117798 |
| 15 | p1 | (A)8 | 14676 | 14683 |  | 102 | p3 | (GAT)4 | 118734 | 118745 |
| 16 | p1 | (A)9 | 14786 | 14794 |  | 103 | p2 | (TC)4 | 120138 | 120145 |
| 17 | c | (AT)6...(A)12 | 16995 | 17058 |  | 104 | p2 | (TC)4 | 120978 | 120985 |
| 18 | p1 | (T)14 | 17163 | 17176 |  | 105 | p2 | (AT)6 | 121250 | 121261 |
| 19 | p2 | (TA)5 | 17378 | 17387 |  | 106 | p1 | (T)11 | 121732 | 121742 |
| 20 | p2 | (TC)4 | 18451 | 18458 |  | 107 | p1 | (C)8 | 122172 | 122179 |
| 21 | c | (T)9...(A)8 | 19657 | 19762 |  | 108 | p1 | (T)9 | 122931 | 122939 |
| 22 | c | (A)8...(ATA)4 | 22593 | 22649 |  | 109 | p1 | (A)8 | 123889 | 123896 |
| 23 | c | (A)13...(T)8 | 24330 | 24421 |  | 110 | p1 | (A)8 | 124843 | 124850 |
| 24 | p1 | (T)8 | 24527 | 24534 |  | 111 | p2 | (AT)4 | 124965 | 124972 |
| 25 | c* | (TC)4(T)10* | 25544 | 25561 |  | 112 | p4 | (TTCT)3 | 125161 | 125172 |
| 26 | p1 | (T)11 | 26377 | 26387 |  | 113 | c | (A)10...(AT)4 | 125463 | 125508 |
| 27 | p2 | (GC)4 | 26937 | 26944 |  | 114 | p1 | (A)8 | 126979 | 126986 |
| 28 | p1 | (T)10 | 28446 | 28455 |  | 115 | p1 | (T)10 | 127114 | 127123 |
| 29 | p1 | (T)8 | 28557 | 28564 |  | 116 | p2 | (CA)4 | 128501 | 128508 |
| 30 | p2 | (TC)5 | 28917 | 28926 |  | 117 | p2 | (TA)5 | 129334 | 129343 |
| 31 | p1 | (A)8 | 32344 | 32351 |  | 118 | p4 | (CTTT)3 | 129772 | 129783 |
| 32 | p3 | (CAA)4 | 38175 | 38186 |  | 119 | p1 | (A)8 | 130194 | 130201 |
| 33 | p1 | (A)8 | 39253 | 39260 |  | 120 | p1 | (A)8 | 131960 | 131967 |
| 34 | p1 | (T)16 | 39596 | 39611 |  | 121 | c | (TA)4...(T)9 | 132381 | 132433 |
| 35 | p1 | (T)8 | 40574 | 40581 |  | 122 | p1 | (T)8 | 132907 | 132914 |
| 36 | p1 | (A)8 | 41673 | 41680 |  | 123 | p1 | (T)9 | 134046 | 134054 |
| 37 | p1 | (G)8 | 44081 | 44088 |  | 124 | p1 | (A)8 | 135208 | 135215 |
| 38 | c | (AT)5...(T)8 | 44975 | 45038 |  | 125 | p2 | (AG)4 | 135508 | 135515 |
| 39 | p2 | (GA)4 | 45151 | 45158 |  | 126 | p1 | (A)8 | 135706 | 135713 |
| 40 | p1 | (A)12 | 45916 | 45927 |  | 127 | p2 | (TA)5 | 137496 | 137505 |
| 41 | p1 | (C)8 | 46239 | 46246 |  | 128 | p1 | (T)9 | 138260 | 138268 |
| 42 | p1 | (C)9 | 46938 | 46946 |  | 129 | p1 | (A)9 | 139211 | 139219 |
| 43 | p2 | (TC)4 | 47870 | 47877 |  | 130 | p1 | (A)8 | 142104 | 142111 |
| 44 | p1 | (C)8 | 49341 | 49348 |  | 131 | c | (T)8...(T)10 | 143370 | 143434 |
| 45 | p1 | (C)14 | 50701 | 50714 |  | 132 | p1 | (A)8 | 143822 | 143829 |
| 46 | p1 | (A)8 | 52831 | 52838 |  | 133 | p1 | (A)9 | 144258 | 144266 |
| 47 | p1 | (A)8 | 54123 | 54130 |  | 134 | p1 | (T)8 | 144539 | 144546 |
| 48 | p1 | (A)11 | 55005 | 55015 |  | 135 | p1 | (T)13 | 145047 | 145059 |
| 49 | p1 | (T)11 | 55262 | 55272 |  | 136 | p1 | (A)8 | 146756 | 146763 |
| 50 | c | (T)13...(TCTT)3 | 55582 | 55638 |  | 137 | p5 | (TTTTC)3 | 146894 | 146908 |
| 51 | c | (TA)4...(T)12...(T)9 | 56896 | 57012 |  | 138 | p1 | (T)9 | 147050 | 147058 |
| 52 | p1 | (A)9 | 57488 | 57496 |  | 139 | c | (TA)4...(AT)4 | 147924 | 147952 |
| 53 | p1 | (A)9 | 58435 | 58443 |  | 140 | p1 | (A)9 | 148462 | 148470 |
| 54 | p1 | (T)11 | 59404 | 59414 |  | 141 | p2 | (TA)5 | 149225 | 149234 |
| 55 | p1 | (T)8 | 60003 | 60010 |  | 142 | p1 | (T)8 | 151017 | 151024 |
| 56 | c* | (C)8(A)9* | 61793 | 61809 |  | 143 | p2 | (CT)4 | 151215 | 151222 |
| 57 | p1 | (T)10 | 62156 | 62165 |  | 144 | p1 | (T)8 | 151515 | 151522 |
| 58 | p3 | (GGT)4 | 62511 | 62522 |  | 145 | p1 | (A)9 | 152676 | 152684 |
| 59 | p4 | (TTCT)3 | 63714 | 63725 |  | 146 | p1 | (A)8 | 153816 | 153823 |
| 60 | p1 | (T)11 | 64352 | 64362 |  | 147 | c | (A)9...(TA)4 | 154297 | 154349 |
| 61 | p1 | (A)8 | 66964 | 66971 |  | 148 | p1 | (T)8 | 154763 | 154770 |
| 62 | p1 | (T)11 | 67589 | 67599 |  | 149 | p1 | (T)8 | 156529 | 156536 |
| 63 | p1 | (G)9 | 68051 | 68059 |  | 150 | p4 | (AAAG)3 | 156947 | 156958 |
| 64 | c | (AT)4...(A)9 | 68291 | 68356 |  | 151 | p2 | (AT)5 | 157386 | 157395 |
| 65 | p1 | (T)8 | 70392 | 70399 |  | 152 | p2 | (TG)4 | 158222 | 158229 |
| 66 | c | (A)8...(CT)4 | 70705 | 70820 |  | 153 | p1 | (A)10 | 159607 | 159616 |
| 67 | p1 | (A)8 | 72126 | 72133 |  | 154 | p1 | (T)8 | 159744 | 159751 |
| 68 | p2 | (CT)5 | 72613 | 72622 |  | 155 | c | (TA)4...(T)10 | 161221 | 161267 |
| 69 | p1 | (T)8 | 72897 | 72904 |  | 156 | p4 | (AAGA)3 | 161557 | 161568 |
| 70 | p1 | (A)9 | 73494 | 73502 |  | 157 | p2 | (AT)4 | 161758 | 161765 |
| 71 | p1 | (A)8 | 73678 | 73685 |  | 158 | p1 | (T)8 | 161880 | 161887 |
| 72 | p1 | (T)9 | 73850 | 73858 |  | 159 | p1 | (T)8 | 162834 | 162841 |
| 73 | p1 | (A)10 | 74451 | 74460 |  | 160 | p1 | (A)9 | 163791 | 163799 |
| 74 | p1 | (T)9 | 74968 | 74976 |  | 161 | p1 | (G)8 | 164551 | 164558 |
| 75 | p3 | (TTG)4 | 75594 | 75605 |  | 162 | p1 | (A)11 | 164988 | 164998 |
| 76 | c | (AT)5...(GA)4 | 77463 | 77545 |  | 163 | p2 | (AT)6 | 165469 | 165480 |
| 77 | c | (AT)8...(AT)4...(GA)4 | 78140 | 78318 |  | 164 | p2 | (GA)4 | 165745 | 165752 |
| 78 | c | (AG)4...(T)9 | 78820 | 78894 |  | 165 | p2 | (GA)4 | 166585 | 166592 |
| 79 | p1 | (T)11 | 79107 | 79117 |  | 166 | p3 | (ATC)4 | 167985 | 167996 |
| 80 | p2 | (AT)4 | 80639 | 80646 |  | 167 | p1 | (A)9 | 168932 | 168940 |
| 81 | p2 | (TA)5 | 80765 | 80774 |  | 168 | p2 | (CT)4 | 170980 | 170987 |
| 82 | c* | (TA)5(AT)4...(AT)9...(TA)4 | 81271 | 81365 |  | 169 | p1 | (A)8 | 171618 | 171625 |
| 83 | p2 | (AT)5 | 84811 | 84820 |  | 170 | p2 | (TC)4 | 174495 | 174502 |
| 84 | p2 | (GA)4 | 86032 | 86039 |  | 171 | p2 | (AG)4 | 176274 | 176281 |
| 85 | p1 | (A)8 | 86406 | 86413 |  | 172 | p1 | (A)8 | 188005 | 188012 |
| 86 | p1 | (A)8 | 87474 | 87481 |  | 173 | p1 | (A)8 | 189471 | 189478 |
| 87 | p1 | (A)9 | 88976 | 88984 |  | 174 | p3 | (TAC)4 | 189755 | 189766 |

**Table S3-3.** Simple repeats (SSR) and their distribution of *C. saxicola* YHL1

| **SSR nr** | **SSR type** | **SSR** | **Start** | **End** |  | | **SSR nr** | **SSR type** | **SSR** | **Start** | **End** |
| --- | --- | --- | --- | --- | --- | --- | --- | --- | --- | --- | --- |
| 1 | c | (A)8...(A)8...(CT)4 | 456 | 622 | |  | 87 | p1 | (T)11 | 91532 | 91542 |
| 2 | p1 | (A)8 | 3388 | 3395 | |  | 88 | p1 | (T)10 | 92589 | 92598 |
| 3 | p1 | (A)9 | 4298 | 4306 | |  | 89 | p2 | (AT)4 | 92833 | 92840 |
| 4 | p1 | (T)10 | 5604 | 5613 | |  | 90 | p1 | (T)12 | 93652 | 93663 |
| 5 | p1 | (T)8 | 7740 | 7747 | |  | 91 | p3 | (GTA)4 | 95255 | 95266 |
| 6 | p2 | (TA)4 | 9109 | 9116 | |  | 92 | p1 | (T)8 | 95543 | 95550 |
| 7 | p1 | (A)8 | 9399 | 9406 | |  | 93 | p1 | (T)8 | 97009 | 97016 |
| 8 | p1 | (A)9 | 9815 | 9823 | |  | 94 | p1 | (A)9 | 102739 | 102747 |
| 9 | c | (T)8...(T)10 | 10339 | 10450 | |  | 95 | p2 | (CT)4 | 108899 | 108906 |
| 10 | p1 | (T)10 | 13871 | 13880 | |  | 96 | p2 | (AG)4 | 110703 | 110710 |
| 11 | p1 | (T)8 | 14016 | 14023 | |  | 97 | p1 | (T)8 | 113581 | 113588 |
| 12 | p1 | (A)8 | 14472 | 14479 | |  | 98 | p2 | (GA)4 | 114218 | 114225 |
| 13 | p3 | (ATT)4 | 14751 | 14762 | |  | 99 | p1 | (T)9 | 116266 | 116274 |
| 14 | p1 | (A)8 | 14949 | 14956 | |  | 100 | p3 | (GAT)4 | 117210 | 117221 |
| 15 | p1 | (A)8 | 15068 | 15075 | |  | 101 | p2 | (TC)4 | 118614 | 118621 |
| 16 | c | (AT)8...(A)11 | 17282 | 17348 | |  | 102 | p2 | (TC)4 | 119460 | 119467 |
| 17 | p1 | (T)10 | 17453 | 17462 | |  | 103 | p2 | (AT)6 | 119732 | 119743 |
| 18 | p2 | (TA)5 | 17664 | 17673 | |  | 104 | p1 | (C)8 | 120651 | 120658 |
| 19 | p2 | (TC)4 | 18737 | 18744 | |  | 105 | p1 | (T)9 | 121410 | 121418 |
| 20 | c | (T)9...(A)8 | 19932 | 20037 | |  | 106 | p1 | (A)8 | 122368 | 122375 |
| 21 | c | (A)8...(ATA)4 | 22874 | 22930 | |  | 107 | p1 | (A)10 | 123319 | 123328 |
| 22 | c | (A)10...(T)8 | 24611 | 24699 | |  | 108 | p2 | (AT)4 | 123443 | 123450 |
| 23 | p1 | (T)9 | 24805 | 24813 | |  | 109 | p4 | (TTCT)3 | 123639 | 123650 |
| 24 | c* | (TC)4(T)9* | 25823 | 25839 | |  | 110 | c | (A)9...(AT)4 | 123941 | 123985 |
| 25 | p1 | (T)13 | 26655 | 26667 | |  | 111 | p1 | (A)8 | 125456 | 125463 |
| 26 | p2 | (GC)4 | 27213 | 27220 | |  | 112 | p1 | (T)11 | 125600 | 125610 |
| 27 | p1 | (T)10 | 28722 | 28731 | |  | 113 | p2 | (CA)4 | 126980 | 126987 |
| 28 | p1 | (T)8 | 28833 | 28840 | |  | 114 | p2 | (TA)5 | 127813 | 127822 |
| 29 | p2 | (TC)5 | 29193 | 29202 | |  | 115 | p4 | (CTTT)3 | 128251 | 128262 |
| 30 | p1 | (A)8 | 32640 | 32647 | |  | 116 | p1 | (A)8 | 128673 | 128680 |
| 31 | p3 | (CAA)6 | 38470 | 38487 | |  | 117 | p1 | (A)8 | 130378 | 130385 |
| 32 | p1 | (A)8 | 39550 | 39557 | |  | 118 | c | (TA)4...(T)9 | 130799 | 130851 |
| 33 | p1 | (T)14 | 39893 | 39906 | |  | 119 | p1 | (T)8 | 131325 | 131332 |
| 34 | p1 | (T)8 | 40869 | 40876 | |  | 120 | p1 | (T)9 | 132464 | 132472 |
| 35 | p1 | (A)8 | 41969 | 41976 | |  | 121 | p1 | (A)8 | 133626 | 133633 |
| 36 | p1 | (G)8 | 44370 | 44377 | |  | 122 | p2 | (AG)4 | 133926 | 133933 |
| 37 | c | (AT)5...(T)8 | 45264 | 45327 | |  | 123 | p1 | (A)8 | 134124 | 134131 |
| 38 | p2 | (GA)4 | 45440 | 45447 | |  | 124 | p2 | (TA)5 | 135914 | 135923 |
| 39 | p1 | (A)11 | 46205 | 46215 | |  | 125 | p1 | (T)9 | 136678 | 136686 |
| 40 | p1 | (C)8 | 46527 | 46534 | |  | 126 | p1 | (A)9 | 137653 | 137661 |
| 41 | p2 | (TC)4 | 47350 | 47357 | |  | 127 | p1 | (T)8 | 139922 | 139929 |
| 42 | p1 | (C)13 | 50182 | 50194 | |  | 128 | p1 | (A)8 | 140551 | 140558 |
| 43 | p1 | (A)8 | 52311 | 52318 | |  | 129 | c | (T)8...(T)10 | 141821 | 141885 |
| 44 | p1 | (A)8 | 53603 | 53610 | |  | 130 | p1 | (A)8 | 142279 | 142286 |
| 45 | p1 | (A)8 | 54485 | 54492 | |  | 131 | p1 | (A)8 | 142715 | 142722 |
| 46 | p1 | (T)10 | 54739 | 54748 | |  | 132 | p1 | (T)8 | 142996 | 143003 |
| 47 | c | (T)14...(TCTT)3 | 55058 | 55115 | |  | 133 | p1 | (T)13 | 143504 | 143516 |
| 48 | c | (TA)4...(T)11...(T)10 | 56373 | 56489 | |  | 134 | p1 | (A)8 | 145195 | 145202 |
| 49 | p1 | (A)9 | 56965 | 56973 | |  | 135 | p5 | (TTTTC)3 | 145333 | 145347 |
| 50 | p1 | (A)8 | 57912 | 57919 | |  | 136 | p1 | (T)9 | 145489 | 145497 |
| 51 | p1 | (T)11 | 58880 | 58890 | |  | 137 | c | (TA)4...(AT)4 | 146363 | 146391 |
| 52 | c | (T)8...(A)8 | 59479 | 59545 | |  | 138 | c | (TA)4...(AT)4 | 146548 | 146576 |
| 53 | c* | (C)8(A)12* | 61264 | 61283 | |  | 139 | p1 | (A)9 | 146923 | 146931 |
| 54 | p3 | (GGT)4 | 61987 | 61998 | |  | 140 | p2 | (TA)5 | 147686 | 147695 |
| 55 | p4 | (TTCT)3 | 63202 | 63213 | |  | 141 | p1 | (T)8 | 149478 | 149485 |
| 56 | p1 | (T)11 | 63840 | 63850 | |  | 142 | p2 | (CT)4 | 149676 | 149683 |
| 57 | p1 | (A)8 | 66452 | 66459 | |  | 143 | p1 | (T)8 | 149976 | 149983 |
| 58 | p1 | (T)9 | 67077 | 67085 | |  | 144 | p1 | (A)9 | 151137 | 151145 |
| 59 | p1 | (G)8 | 67537 | 67544 | |  | 145 | p1 | (A)8 | 152277 | 152284 |
| 60 | c | (AT)4...(A)11 | 67776 | 67843 | |  | 146 | c | (A)9...(TA)4 | 152758 | 152810 |
| 61 | p1 | (T)8 | 69874 | 69881 | |  | 147 | p1 | (T)8 | 153224 | 153231 |
| 62 | c | (A)8...(CT)4 | 70187 | 70302 | |  | 148 | p1 | (T)8 | 154929 | 154936 |
| 63 | p1 | (A)8 | 71607 | 71614 | |  | 149 | p4 | (AAAG)3 | 155347 | 155358 |
| 64 | p2 | (CT)5 | 72094 | 72103 | |  | 150 | p2 | (AT)5 | 155786 | 155795 |
| 65 | p1 | (T)8 | 72378 | 72385 | |  | 151 | p2 | (TG)4 | 156622 | 156629 |
| 66 | p1 | (A)9 | 72975 | 72983 | |  | 152 | p1 | (A)11 | 157999 | 158009 |
| 67 | p1 | (A)8 | 73165 | 73172 | |  | 153 | p1 | (T)8 | 158146 | 158153 |
| 68 | p1 | (T)9 | 73337 | 73345 | |  | 154 | c | (TA)4...(T)9 | 159643 | 159688 |
| 69 | p1 | (A)9 | 73938 | 73946 | |  | 155 | p4 | (AAGA)3 | 159978 | 159989 |
| 70 | p1 | (T)9 | 74454 | 74462 | |  | 156 | p2 | (AT)4 | 160179 | 160186 |
| 71 | p3 | (TTG)4 | 75075 | 75086 | |  | 157 | p1 | (T)10 | 160301 | 160310 |
| 72 | p2 | (GA)4 | 77200 | 77207 | |  | 158 | p1 | (T)8 | 161254 | 161261 |
| 73 | c | (AG)4...(T)9 | 77710 | 77784 | |  | 159 | p1 | (A)9 | 162211 | 162219 |
| 74 | p1 | (T)11 | 77997 | 78007 | |  | 160 | p1 | (G)8 | 162971 | 162978 |
| 75 | p2 | (AT)4 | 79523 | 79530 | |  | 161 | p2 | (AT)6 | 163886 | 163897 |
| 76 | p2 | (TA)5 | 79649 | 79658 | |  | 162 | p2 | (GA)4 | 164162 | 164169 |
| 77 | c* | (T)8(AT)9...(AT)9...(TA)7 | 79784 | 79900 | |  | 163 | p2 | (GA)4 | 165008 | 165015 |
| 78 | c | (AT)5...(AT)5 | 83557 | 83662 | |  | 164 | p3 | (ATC)4 | 166408 | 166419 |
| 79 | p2 | (TA)8 | 84174 | 84189 | |  | 165 | p1 | (A)9 | 167355 | 167363 |
| 80 | p2 | (GA)4 | 84304 | 84311 | |  | 166 | p2 | (CT)4 | 169403 | 169410 |
| 81 | p1 | (A)8 | 84678 | 84685 | |  | 167 | p1 | (A)8 | 170041 | 170048 |
| 82 | p1 | (A)8 | 85746 | 85753 | |  | 168 | p2 | (AG)4 | 174716 | 174723 |
| 83 | p1 | (A)8 | 87254 | 87261 | |  | 169 | p1 | (A)8 | 186805 | 186812 |
| 84 | p2 | (AT)4 | 89504 | 89511 | |  | 170 | p1 | (A)8 | 188271 | 188278 |
| 85 | c | (T)8...(T)9 | 89930 | 89961 | |  | 171 | p3 | (TAC)4 | 188555 | 188566 |
| 86 | p1 | (T)13 | 91097 | 91109 | |  |  |  |  |  |  |

**Table S3-4.** Simple repeats (SSR) and their distribution of *C.saxicola* YHL2

| **SSR nr** | **SSR type** | **SSR** | **Start** | **End** |  | | **SSR nr** | **SSR type** | **SSR** | **Start** | **End** |
| --- | --- | --- | --- | --- | --- | --- | --- | --- | --- | --- | --- |
| 1 | c | (A)8...(A)8...(CT)4 | 455 | 621 | |  | 86 | p1 | (T)13 | 91322 | 91334 |
| 2 | p1 | (A)8 | 3387 | 3394 | |  | 87 | p1 | (T)11 | 91757 | 91767 |
| 3 | p1 | (A)9 | 4297 | 4305 | |  | 88 | p1 | (T)10 | 92814 | 92823 |
| 4 | p1 | (T)10 | 5603 | 5612 | |  | 89 | p1 | (T)12 | 93902 | 93913 |
| 5 | p1 | (T)8 | 7739 | 7746 | |  | 90 | p3 | (GTA)4 | 95505 | 95516 |
| 6 | p2 | (TA)4 | 9108 | 9115 | |  | 91 | p1 | (T)8 | 95793 | 95800 |
| 7 | p1 | (A)8 | 9398 | 9405 | |  | 92 | p1 | (T)8 | 97259 | 97266 |
| 8 | p1 | (A)9 | 9814 | 9822 | |  | 93 | p1 | (A)9 | 103015 | 103023 |
| 9 | c | (T)8...(T)10 | 10338 | 10449 | |  | 94 | p2 | (CT)4 | 109174 | 109181 |
| 10 | p1 | (T)10 | 13870 | 13879 | |  | 95 | p2 | (AG)4 | 110978 | 110985 |
| 11 | p1 | (T)8 | 14015 | 14022 | |  | 96 | p1 | (T)8 | 113856 | 113863 |
| 12 | p1 | (A)8 | 14471 | 14478 | |  | 97 | p2 | (GA)4 | 114493 | 114500 |
| 13 | p3 | (ATT)4 | 14750 | 14761 | |  | 98 | p1 | (T)9 | 116541 | 116549 |
| 14 | p1 | (A)8 | 14948 | 14955 | |  | 99 | p3 | (GAT)4 | 117485 | 117496 |
| 15 | p1 | (A)8 | 15067 | 15074 | |  | 100 | p2 | (TC)4 | 118889 | 118896 |
| 16 | c | (AT)8...(A)11 | 17281 | 17347 | |  | 101 | p2 | (TC)4 | 119735 | 119742 |
| 17 | p1 | (T)10 | 17452 | 17461 | |  | 102 | p2 | (AT)6 | 120007 | 120018 |
| 18 | p2 | (TA)5 | 17663 | 17672 | |  | 103 | p1 | (C)8 | 120926 | 120933 |
| 19 | p2 | (TC)4 | 18736 | 18743 | |  | 104 | p1 | (T)9 | 121685 | 121693 |
| 20 | c | (T)9...(A)8 | 19931 | 20036 | |  | 105 | p1 | (A)8 | 122643 | 122650 |
| 21 | c | (A)8...(ATA)4 | 22873 | 22929 | |  | 106 | p1 | (A)10 | 123594 | 123603 |
| 22 | c | (A)10...(T)8 | 24610 | 24698 | |  | 107 | p2 | (AT)4 | 123718 | 123725 |
| 23 | p1 | (T)9 | 24804 | 24812 | |  | 108 | p4 | (TTCT)3 | 123914 | 123925 |
| 24 | c* | (TC)4(T)9* | 25822 | 25838 | |  | 109 | c | (A)9...(AT)4 | 124216 | 124260 |
| 25 | p1 | (T)13 | 26654 | 26666 | |  | 110 | p1 | (A)8 | 125731 | 125738 |
| 26 | p2 | (GC)4 | 27212 | 27219 | |  | 111 | p1 | (T)11 | 125875 | 125885 |
| 27 | p1 | (T)10 | 28721 | 28730 | |  | 112 | p2 | (CA)4 | 127255 | 127262 |
| 28 | p1 | (T)8 | 28832 | 28839 | |  | 113 | p2 | (TA)5 | 128088 | 128097 |
| 29 | p2 | (TC)5 | 29192 | 29201 | |  | 114 | p4 | (CTTT)3 | 128526 | 128537 |
| 30 | p1 | (A)8 | 32640 | 32647 | |  | 115 | p1 | (A)8 | 128948 | 128955 |
| 31 | p3 | (CAA)6 | 38470 | 38487 | |  | 116 | p1 | (A)8 | 130653 | 130660 |
| 32 | p1 | (A)8 | 39550 | 39557 | |  | 117 | c | (TA)4...(T)9 | 131074 | 131126 |
| 33 | p1 | (T)14 | 39893 | 39906 | |  | 118 | p1 | (T)8 | 131600 | 131607 |
| 34 | p1 | (T)8 | 40869 | 40876 | |  | 119 | p1 | (T)9 | 132739 | 132747 |
| 35 | p1 | (A)8 | 41969 | 41976 | |  | 120 | p1 | (A)8 | 133901 | 133908 |
| 36 | p1 | (G)8 | 44370 | 44377 | |  | 121 | p2 | (AG)4 | 134201 | 134208 |
| 37 | c | (AT)5...(T)8 | 45264 | 45327 | |  | 122 | p1 | (A)8 | 134399 | 134406 |
| 38 | p2 | (GA)4 | 45440 | 45447 | |  | 123 | p2 | (TA)5 | 136189 | 136198 |
| 39 | p1 | (A)11 | 46205 | 46215 | |  | 124 | p1 | (T)9 | 136953 | 136961 |
| 40 | p1 | (C)8 | 46527 | 46534 | |  | 125 | p1 | (A)9 | 137930 | 137938 |
| 41 | p2 | (TC)4 | 47416 | 47423 | |  | 126 | p1 | (T)8 | 140199 | 140206 |
| 42 | p1 | (C)13 | 50248 | 50260 | |  | 127 | p1 | (A)8 | 140828 | 140835 |
| 43 | p1 | (A)8 | 52377 | 52384 | |  | 128 | c | (T)8...(T)10 | 142094 | 142158 |
| 44 | p1 | (A)8 | 53669 | 53676 | |  | 129 | p1 | (A)8 | 142552 | 142559 |
| 45 | p1 | (A)8 | 54551 | 54558 | |  | 130 | p1 | (A)8 | 142988 | 142995 |
| 46 | p1 | (T)10 | 54805 | 54814 | |  | 131 | p1 | (T)8 | 143269 | 143276 |
| 47 | c | (T)14...(TCTT)3 | 55124 | 55181 | |  | 132 | p1 | (T)13 | 143777 | 143789 |
| 48 | c | (TA)4...(T)11...(T)10 | 56439 | 56555 | |  | 133 | p1 | (A)8 | 145468 | 145475 |
| 49 | p1 | (A)9 | 57031 | 57039 | |  | 134 | p5 | (TTTTC)3 | 145606 | 145620 |
| 50 | p1 | (A)8 | 57978 | 57985 | |  | 135 | p1 | (T)9 | 145762 | 145770 |
| 51 | p1 | (T)11 | 58946 | 58956 | |  | 136 | c | (TA)4...(AT)4 | 146636 | 146664 |
| 52 | c | (T)8...(A)8 | 59545 | 59611 | |  | 137 | c | (TA)4...(AT)4 | 146821 | 146849 |
| 53 | c* | (C)8(A)12* | 61330 | 61349 | |  | 138 | p1 | (A)9 | 147196 | 147204 |
| 54 | p3 | (GGT)4 | 62053 | 62064 | |  | 139 | p2 | (TA)5 | 147959 | 147968 |
| 55 | p4 | (TTCT)3 | 63268 | 63279 | |  | 140 | p1 | (T)8 | 149751 | 149758 |
| 56 | p1 | (T)11 | 63906 | 63916 | |  | 141 | p2 | (CT)4 | 149949 | 149956 |
| 57 | p1 | (A)8 | 66518 | 66525 | |  | 142 | p1 | (T)8 | 150249 | 150256 |
| 58 | p1 | (T)9 | 67143 | 67151 | |  | 143 | p1 | (A)9 | 151410 | 151418 |
| 59 | p1 | (G)8 | 67603 | 67610 | |  | 144 | p1 | (A)8 | 152550 | 152557 |
| 60 | c | (AT)4...(A)11 | 67842 | 67909 | |  | 145 | c | (A)9...c(TA)4 | 153031 | 153083 |
| 61 | p1 | (T)8 | 69940 | 69947 | |  | 146 | p1 | (T)8 | 153497 | 153504 |
| 62 | c | (A)8...(CT)4 | 70253 | 70368 | |  | 147 | p1 | (T)8 | 155202 | 155209 |
| 63 | p1 | (A)8 | 71673 | 71680 | |  | 148 | p4 | (AAAG)3 | 155620 | 155631 |
| 64 | p2 | (CT)5 | 72160 | 72169 | |  | 149 | p2 | (AT)5 | 156059 | 156068 |
| 65 | p1 | (T)8 | 72444 | 72451 | |  | 150 | p2 | (TG)4 | 156895 | 156902 |
| 66 | p1 | (A)9 | 73041 | 73049 | |  | 151 | p1 | (A)11 | 158272 | 158282 |
| 67 | p1 | (A)8 | 73231 | 73238 | |  | 152 | p1 | (T)8 | 158419 | 158426 |
| 68 | p1 | (T)9 | 73403 | 73411 | |  | 153 | c | (TA)4...(T)9 | 159916 | 159961 |
| 69 | p1 | (A)9 | 74004 | 74012 | |  | 154 | p4 | (AAGA)3 | 160251 | 160262 |
| 70 | p1 | (T)9 | 74520 | 74528 | |  | 155 | p2 | (AT)4 | 160452 | 160459 |
| 71 | p3 | (TTG)4 | 75141 | 75152 | |  | 156 | p1 | (T)10 | 160574 | 160583 |
| 72 | p2 | (GA)4 | 77364 | 77371 | |  | 157 | p1 | (T)8 | 161527 | 161534 |
| 73 | c | (AG)4...(T)9 | 77934 | 78008 | |  | 158 | p1 | (A)9 | 162484 | 162492 |
| 74 | p1 | (T)11 | 78221 | 78231 | |  | 159 | p1 | (G)8 | 163244 | 163251 |
| 75 | p2 | (AT)4 | 79747 | 79754 | |  | 160 | p2 | (AT)6 | 164159 | 164170 |
| 76 | p2 | (TA)5 | 79873 | 79882 | |  | 161 | p2 | (GA)4 | 164435 | 164442 |
| 77 | c* | (T)8(AT)9*...(AT)9...(TA)7 | 80008 | 80124 | |  | 162 | p2 | (GA)4 | 165281 | 165288 |
| 78 | c | (AT)5...(AT)5 | 83782 | 83887 | |  | 163 | p3 | (ATC)4 | 166681 | 166692 |
| 79 | p2 | (TA)8 | 84399 | 84414 | |  | 164 | p1 | (A)9 | 167628 | 167636 |
| 80 | p2 | (GA)4 | 84529 | 84536 | |  | 165 | p2 | (CT)4 | 169676 | 169683 |
| 81 | p1 | (A)8 | 84903 | 84910 | |  | 166 | p1 | (A)8 | 170314 | 170321 |
| 82 | p1 | (A)8 | 85971 | 85978 | |  | 167 | p2 | (AG)4 | 174997 | 175004 |
| 83 | p1 | (A)8 | 87479 | 87486 | |  | 168 | p1 | (A)8 | 187183 | 187190 |
| 84 | p2 | (AT)4 | 89729 | 89736 | |  | 169 | p1 | (A)8 | 188649 | 188656 |
| 85 | c | (T)8...(T)9 | 90155 | 90186 | |  | 170 | p3 | (TAC)4 | 188933 | 188944 |
